# Dissecting the Cell‐Type‐Specific Response to an Emerging Tobamovirus in Tomato Reveals Cultivar‐Dependent Involvement of Brassinosteroid Signalling

**DOI:** 10.1111/pbi.70559

**Published:** 2026-01-21

**Authors:** Yuhong Zhang, Shan Bu, Yuxin Nie, Luyou Wang, Jiayi Liu, Junchen Xu, Jiejun Peng, Fei Yan, Jian Wu

**Affiliations:** ^1^ State Key Laboratory for Managing Biotic and Chemical Threats to the Quality and Safety of Agro‐Products, Key Laboratory of Biotechnology in Plant Protection of MARA, Key Laboratory of Green Plant Protection of Zhejiang Province, Institute of Plant Virology Ningbo University Ningbo China

**Keywords:** brassinosteroid signalling, cultivar, single‐cell seq, tomato, tomato brown rugose fruit virus

## Abstract

Plant viruses drive widespread crop epidemics, yet the host plant responses across different cell types, particularly how these responses are influenced by cultivars with varying genetic backgrounds, including the presence of resistance (R) genes, remain poorly understood. Using tomato brown rugose fruit virus (ToBRFV) and two tomato cultivars, ‘Jinpeng No. 1’ (JP) and ‘Rutgers’ (RG), with different genetic backgrounds, this study used single‐cell RNA sequencing to explore infection dynamics and responses at the cellular level. Results showed that ToBRFV accumulated to different levels in the two cultivars, likely due to differences in their genetic backgrounds, particularly the distinct genotypes of the *Tm‐2* ^*2*^ and *tm‐2* alleles. Following infection, the composition of cell types in tomato leaves also varied between the two cultivars. While the entry or movement of ToBRFV in the JP cultivar was not fully prevented early on, the viral accumulation in certain cell types of this cultivar was restricted. ToBRFV alters signalling pathways based on cell type and cultivars. Pseudotime analysis revealed that, in JP plants, ToBRFV reverses expression of brassinosteroid (BR) positive regulators during mesophyll cell development. Silencing positive BR regulators increased infection in JP plants, while suppressing it in RG plants, linking BR signalling to JP‐dependent resistance. Exogenous BR suppressed ToBRFV in JP but enhanced it in RG plants. This study reveals the differential involvement of BR signalling during viral infection in the two cultivars, offering a framework for future studies of plant‐virus interactions.

## 1 Introduction

Plant viruses are major pathogens that cause significant damage to crops worldwide, leading to severe yield losses and threatening global food security (Jones 2021). To combat these viral infections, plants have evolved sophisticated defence mechanisms, among which resistance (R) genes play a pivotal role (Sett et al. 2022). R genes typically confer resistance via two mechanisms: active resistance, wherein R proteins recognise viral avirulence factors and trigger a hypersensitive response (HR) characterised by localised cell death to limit viral movement (Sett et al. 2022); and passive resistance, which involves recessive alleles encoding host factors essential for viral infection, such as eukaryotic translation initiation factors, alterations of which prevent viral establishment (Kourelis and Van Der Hoorn 2018). To date, the identification of R genes and the characterisation of their role in plant‐virus interactions remain fundamental to antiviral breeding.

Our understanding of plant‐virus interactions has primarily been shaped by bulk tissue analyses. However, plant tissues contain diverse cell types with distinct metabolic activities, signalling pathways, and chemical environments, shaping cell‐type‐specific gene expression, including R genes (Kitashova et al. 2023). R gene‐mediated antiviral pathways may also vary among cell types due to differences in receptor levels, signalling components, and metabolic conditions (Venkatesh and Kang 2019). To date, it remains unclear whether the R gene functions differently across various cell types. Single‐cell RNA sequencing (scRNA‐seq) enables transcriptomic profiling at the single‐cell level, revealing cellular heterogeneity and gene functions, such as R genes, in response to viral attack. It uncovers plant‐virus interactions and immune responses that bulk tissue analyses miss (Serrano et al. 2024; Zhu, Moreno‐Pérez, and Coaker 2023).

In addition to the inherent cellular diversity within plants, different cultivars of the same species may possess distinct genetic backgrounds that influence their interaction with plant viruses. For instance, the *Tm‐2* ^*2*^, *Tm‐2*, and *tm‐2* alleles, all located at the same genetic locus in tomato plants, demonstrate varying responses to viral threats. *Tm‐2* ^*2*^ and *Tm‐2* are R genes that produce CC‐NBS‐LRR proteins that resist tobamoviruses, such as tobacco mosaic virus (TMV) and tomato mosaic virus (ToMV), by recognising viral movement proteins (MPs) (Hak et al. 2023; Hak and Spiegelman 2021). *Tm‐2* ^*2*^ provides more durable, broad‐spectrum resistance than *Tm‐2* (Hall 1980; Lanfermeijer et al. 2005; Tettey et al. 2023), while the *tm‐2* allele offers no resistance, making plants susceptible (Shi et al. 2011). However, the newly emerged tomato brown rugose fruit virus (ToBRFV) has overcome this defence by evading the *Tm‐2* ^*2*^‐mediated immune response, even though its MP may incur a fitness cost, thus preventing reliable resistance in *Tm‐2* ^*2*^ cultivars (Hak et al. 2023; Hak and Spiegelman 2021). Consequently, identifying novel R genes is crucial for developing tomato cultivars with durable resistance to ToBRFV. However, it is important to carefully consider whether R genes identified in one cultivar can be effectively applied to other cultivars with distinct genetic backgrounds, such as variations in the presence of *Tm‐2* ^*2*^, *Tm‐2*, and *tm‐2* alleles.

In this study, the tomato cultivars ‘Jinpeng No. 1’ (JP, *Tm‐2* ^*2*^/*tm‐2*) and ‘Rutgers’ (RG, *tm‐2*/*tm‐2*) were analysed using scRNA‐seq, molecular, and microscopic techniques to explore their cell‐type‐specific and cultivar‐specific responses to ToBRFV infection. Our findings showed that cell‐type composition, viral accumulation, and host signalling varied both among cell types and between JP and RG during infection. Notably, we observed opposing roles of brassinosteroid (BR) signalling in the two cultivars in response to ToBRFV infection. Specifically, the activation of BR signalling in the JP cultivar may serve as a potential target for suppressing ToBRFV infection. This work offers the first cell‐type‐resolved characterisation of viral infection in two tomato cultivars, highlighting the complex interplay among the tomato cultivar, ToBRFV and BR signalling. These findings provide new insights into how genetic background and cell‐specific signalling pathways shape the response to viral infections, which could inform future strategies for breeding more resistant tomato varieties.

## 2 Results

### 2.1 ToBRFV Accumulated to Relatively Lower Levels in JP Cultivar Compared to RG Cultivar

**FIGURE 1:**
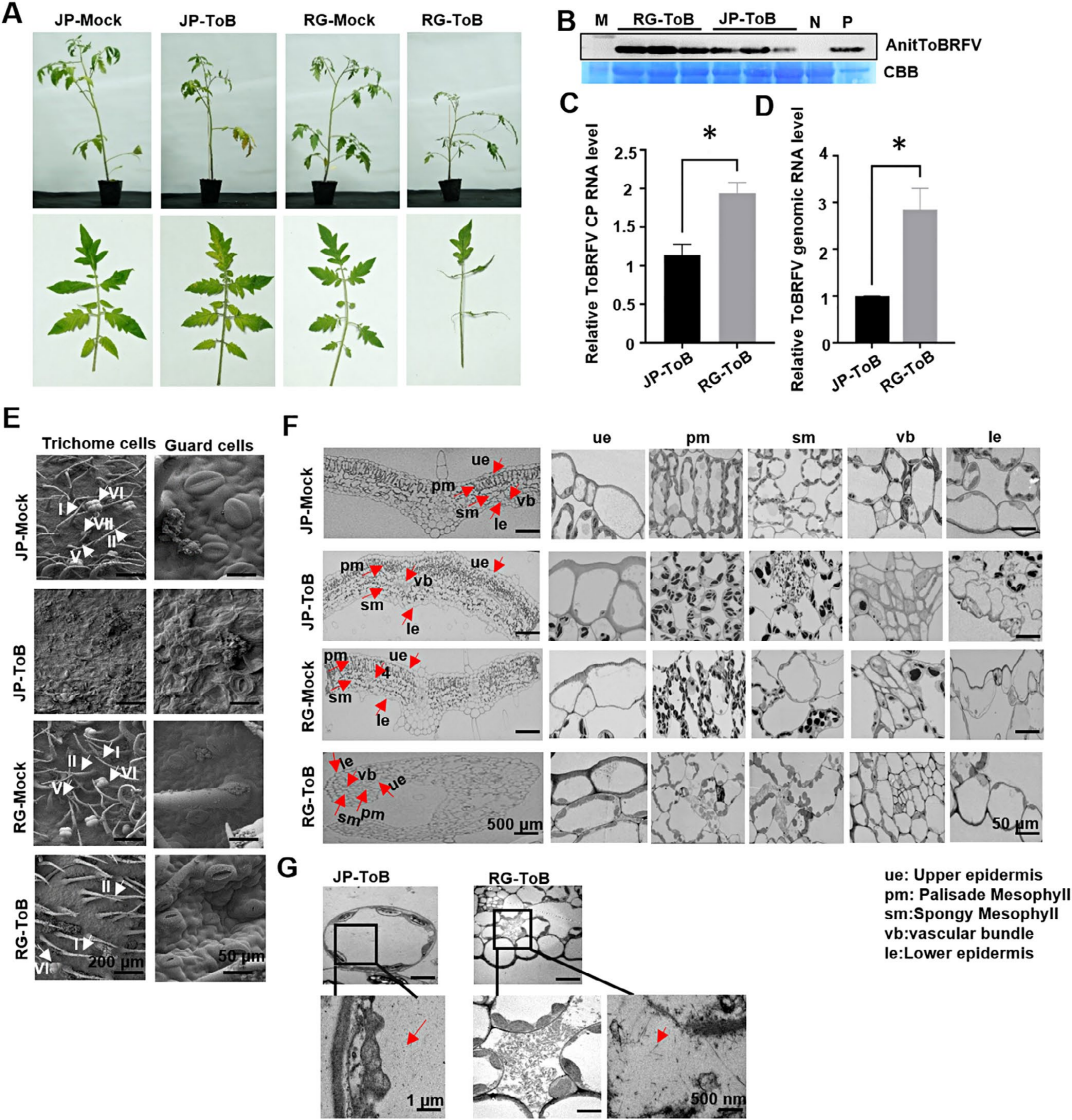
The infection of ToBRFV was mitigated in JP cultivars. (A) Disease symptoms observed in RG (cv. ‘Rutgers’) or the JP (cv. ‘Jinpeng’) tomato plants at 14 days post‐infection (dpi) with ToBRFV. Plants were agroinfiltrated with a ToBRFV infectious clone, and mock‐inoculated controls received Agrobacterium harbouring the pCB301‐GUS vector. Leaf curling and wiry morphology were prominent in infected RG plants (RG‐ToB), while infected JP plants (JP‐ToB) exhibited milder symptoms, including mosaic patterns and necrotic spots on lower leaves. No symptoms were observed in mock‐inoculated plants (RG‐Mock and JP‐Mock). Three biological replicates were performed (four plants per replicate); data from a representative replicate are shown. (B) Western blot detection of ToBRFV coat protein (CP) in systemic leaves at 14 dpi using anti‐CP antibody. Coomassie Brilliant Blue (CBB) staining of Rubisco large subunit (RbcL) served as the loading control. A CP‐positive sample was included as a positive control (P). (C, D) QRT‐PCR analysis of ToBRFV CP sub‐genomic (C) and genomic RNA (D) accumulation in infected RG and JP plants was performed at 14 dpi. Data were analysed using Student's *t*‐test. *, *p* < 0.05. (E) Cryo‐scanning electron microscopy (cryo‐SEM) of trichomes and guard cells. Type I, II, V, and VI‐like trichomes were observed in both mock‐inoculated RG and JP plants, while ToBRFV infection in RG plants resulted in predominantly type I‐, II‐ and VI‐like trichomes. Additionally, type VII‐like trichomes were observed in JP‐Mock plants. Dehydrated trichomes and guard cells were observed in symptomatic JP‐ToB leaves. ToBRFV infection promoted stomatal opening in RG‐ToB plants compared to mock controls. The scale bars represent 200 μm and 50 μm for observations of trichomes and guard cells, respectively. (F) Cryo‐SEM of cross‐sections through the midrib showing tissue architecture: Upper epidermis (ue), palisade mesophyll (pm), spongy mesophyll (sm), vascular bundle (vb), and lower epidermis (le). Infected RG and JP plants displayed irregularly shaped palisade mesophyll cells, unlike the cylindrical, perpendicular arrangement seen in mock plants. Cell rupture was apparent in palisade mesophyll of RG‐ToB plants and spongy mesophyll of both RG‐ToB and JP‐ToB plants. Altered morphology of lower epidermal cells was noted in JP‐ToB plants. Scale bars = 500 μm or 50 μm. (G) Transmission electron microscopy (TEM) of leaf blade sections revealed ToBRFV virions (red arrows) localised within ruptured spongy mesophyll cells near the lower epidermis in both RG‐ToB and JP‐ToB plants. Scale bar = 500 nm.

To analyse the infection of ToBRFV in two cultivars, Agrobacterium containing the ToBRFV cDNA infectious clone was inoculated into plants. Negative controls included plants inoculated with the pCB301‐GUS construct. Each treatment had three biological replicates of four plants. At 14 days post‐infection (dpi), systemic leaves of RG plants inoculated with ToBRFV (RG‐ToB) exhibited characteristic wiry and curled symptoms, whereas ToBRFV‐infected JP plants (JP‐ToB) displayed significantly milder symptoms. This indicates that while JP is susceptible to ToBRFV, the severity of the symptoms is significantly less than that observed in RG. No symptoms were seen in mock‐inoculated plants. JP‐ToB plants displayed sporadic necrotic lesions and mosaic patterns in lower leaves, which were absent in RG‐ToB plants (Figure 1A).

At 14 dpi, western blot analysis revealed detectable levels of ToBRFV coat protein (CP) in all infected plants, with significantly lower CP levels observed in JP‐ToB plants compared to RG‐ToB plants (Figure 1B). Consistently, qRT‐PCR showed that ToBRFV CP RNA (Figure 1C) and genomic RNA (Figure 1D) levels were significantly lower in JP‐ToB plants compared to RG‐ToB plants. These findings suggest that ToBRFV infection is limited in the JP cultivar.

Cryo‐scanning electron microscopy (cryo‐SEM) was performed on leaves exhibiting severe disease symptoms at 21 dpi to examine morphological changes in trichomes and guard cells. Leaf surface of RG‐Mock, JP‐Mock, and RG‐ToB plants exhibited typical trichome types and stomatal morphology (Kang et al. 2010; Zhang et al. 2020), while JP‐ToB leaves lacked typical trichomes. Types I, II, V, and VI trichomes appeared in both mock plants, with type VII also present in JP‐Mock; RG‐ToB mainly showed types I, II, and VI. Additionally, ToBRFV induced increased stomatal opening in RG plants compared to mock controls (Figure 1E).

Cryo‐SEM revealed pathological changes in the palisade and spongy mesophyll layers of infected RG and JP plants. Infected palisade cells were irregular and circular, unlike the cylindrical, perpendicular shape in mock plants. Cell rupture was seen in RG‐ToB palisade cells. The spongy mesophyll showed irregular morphology and cell rupture in both RG and JP plants. Additionally, lower epidermal cells in JP‐ToB plants exhibited shape alterations (Figure 1F). Transmission electron microscopy (TEM) further revealed virion cross sections localised within ruptured spongy mesophyll cells near the lower epidermis in both RG‐ToB and JP‐ToB samples (Figure 1G), underscoring the cellular impact of ToBRFV infection.

### 2.2 ScRNA‐Seq Analysis Reveals Different Tomato Leaf Cell‐Type Diversity in JP and RG Two Cultivars During ToBRFV Infection

**FIGURE 2:**
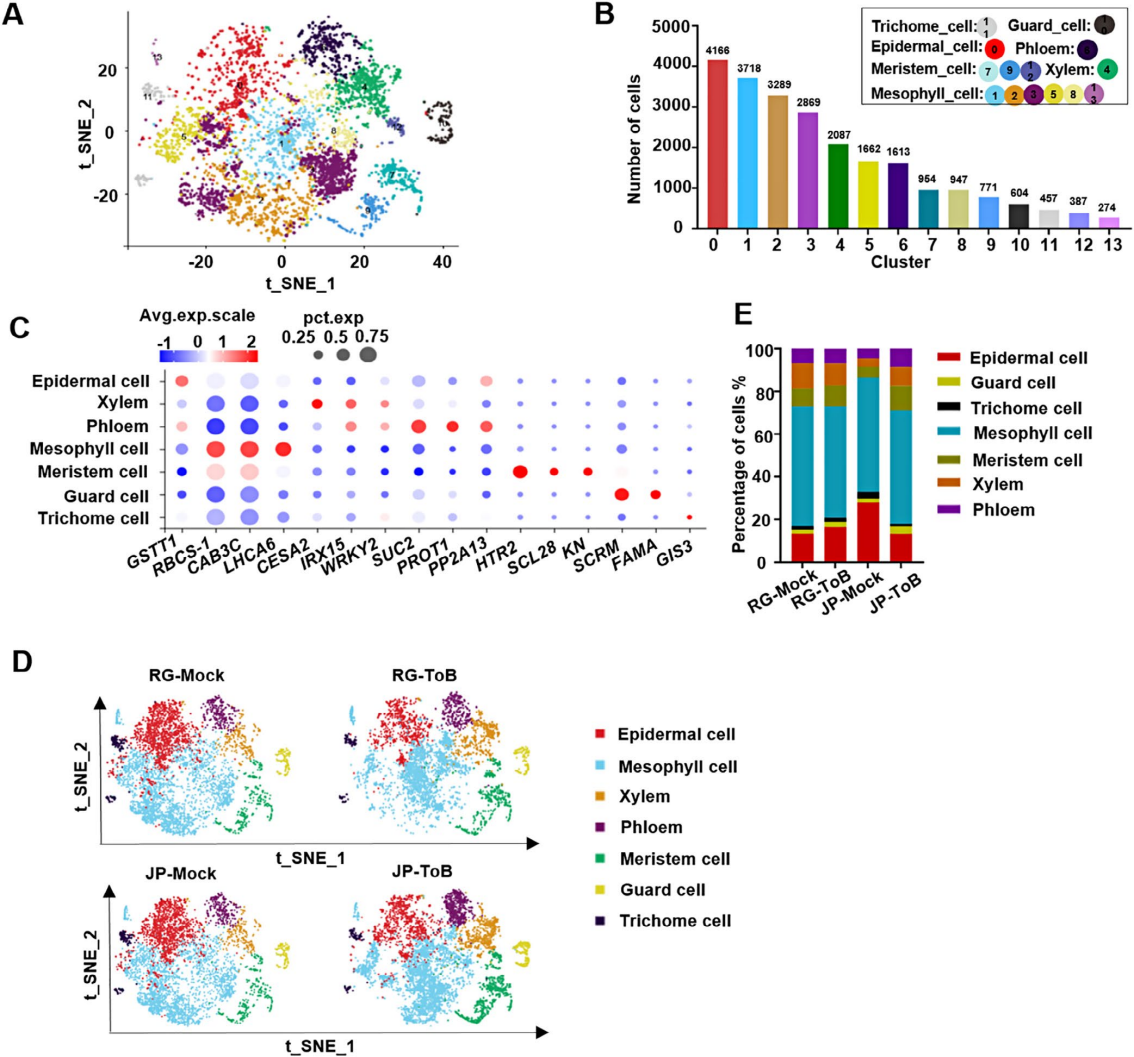
Single‐cell transcriptomic profiling reveals different tomato leaf cell‐type diversity in JP and RG two cultivars during ToBRFV infection. (A) t‐SNE visualisation of 27 094 cells isolated from systemic leaves of tomato plants across four groups (JP‐Mock, RG‐Mock, JP‐ToB, RG‐ToB), grouped into 14 transcriptionally distinct clusters. Each dot represents a single cell, and each colour indicates a unique cluster. (B) Identification of seven major cell types: epidermal cells (cluster 0), trichome cells (cluster 11), guard cells (cluster 10), mesophyll cells (clusters 1, 2, 3, 5, 8, 13), meristem cells (clusters 7, 9, 12), xylem cells (cluster 4), and phloem cells (cluster 6), based on expression of Arabidopsis homologous marker genes. (C) Dot plot showing the expression patterns of representative marker genes used to assign cell identity for each cluster. Dot size corresponds to the percentage of cells within a cluster expressing a given gene, and colour intensity indicates average expression level. (D) t‐SNE projections of cell populations from mock‐inoculated and ToBRFV‐infected RG and JP plants. JP and RG mock plants exhibited similar global topologies, while ToBRFV infection induced distinct shifts in cellular distribution, particularly in mesophyll and epidermal populations. (E) Bar plot showing the proportions of each cell type across cultivars and treatments.

To analyse ToBRFV infection in two cultivars at the cellular level, scRNA‐seq was performed on the youngest leaves at 14 dpi, profiling 8522 cells (JP‐ToB), 6043 cells (RG‐ToB), 6487 cells (JP‐Mock), and 7052 cells (RG‐Mock), detecting 2524–3238 genes per cell (Table S1 and Figure S1A). Clustering analysis identified 14 distinct cell clusters via t‐SNE (Figure 2A), representing 0.16% to 28.03% of total cells (Table S2 and Figure S1B). The distribution of key quality metrics per cell was presented (Figure S1C). Cluster annotation was based on *Arabidopsis thaliana* marker gene homologues, classifying the clusters into seven major cell types: epidermal, guard, trichome, mesophyll, meristem, xylem, and phloem (Figure 2B,C) (Brown et al. 2011; Burn et al. 2002; Guillaumie et al. 2010; Kallemi et al. 2024; Lang et al. 2016; Liu et al. 2021; Long et al. 1996; Nomoto et al. 2022; Park et al. 2015; Pichersky et al. 1985; Sawchuk et al. 2008; Seo et al. 2022; Shirakawa et al. 2014; Sun et al. 2015; Wei et al. 2020; Yang et al. 2023; Yin et al. 2014; Yue et al. 2024). For each cell type, gene sets were refined by identifying genes with significantly higher expression than others (Table S3), confirming tomato leaf cellular heterogeneity. The top five expressed genes per cluster were visualised in a heatmap (Figure S2A), and the 14 highest‐expressed genes per cell type were mapped on the t‐SNE plot (Figure S2B).

To examine the cellular heterogeneity of tomato leaf responses to ToBRFV, t‐SNE projections were generated for RG‐Mock and JP‐Mock samples, showing similar overall topologies (Figure 2D). However, significant differences in cell type proportions were observed. JP‐Mock plants had a notable increase in epidermal cells (from 13.32% to 28.03%) and a decrease in xylem cells (from 11.93% to 3.84%) compared to RG‐Mock plants. Meristem and phloem cells showed modest reductions in JP‐Mock plants (Table S4 and Figure 2E).

Subsequently, scRNA‐seq profiles of RG and JP tomato leaves following ToBRFV infection were characterised. Infected samples showed similar t‐SNE topology to their mock controls, except for notable changes in mesophyll cells (Figure 2D). Comparative analysis of cell‐type proportions revealed distinct responses to ToBRFV based on cultivars. In RG‐ToB plants, epidermal cells increased from 13.32% to 16.53%, while in JP‐ToB plants, they decreased from 28.03% to 13.23%. JP‐ToB plants also showed increases in xylem (3.84%–9.07%), phloem (4.50%–8.46%), and meristem cells (5.29%–11.50%) compared to JP‐Mock plants (Table S4 and Figure 2E). In contrast, proportions of xylem, phloem, and meristem cells were similar between RG‐ToB and RG‐Mock plants. These findings indicate that ToBRFV infection differentially modulates cell differentiation in two cultivars.

ScRNA‐seq showed similar trichome cell proportions in the youngest systemic leaves of JP‐ToB and RG‐ToB plants (Figure 2E), while cryo‐SEM revealed no typical trichomes in lower JP‐ToB leaves with severe symptoms (Figure 1E). These results, from different time points (14 vs. 21 dpi) and leaves, suggest a gradual loss of trichome cells during leaf development under prolonged ToBRFV infection.

### 2.3 Cell Type‐Specific Modulation of ToBRFV Accumulation in RG and JP Tomato Plants During Early Infection

**FIGURE 3:**
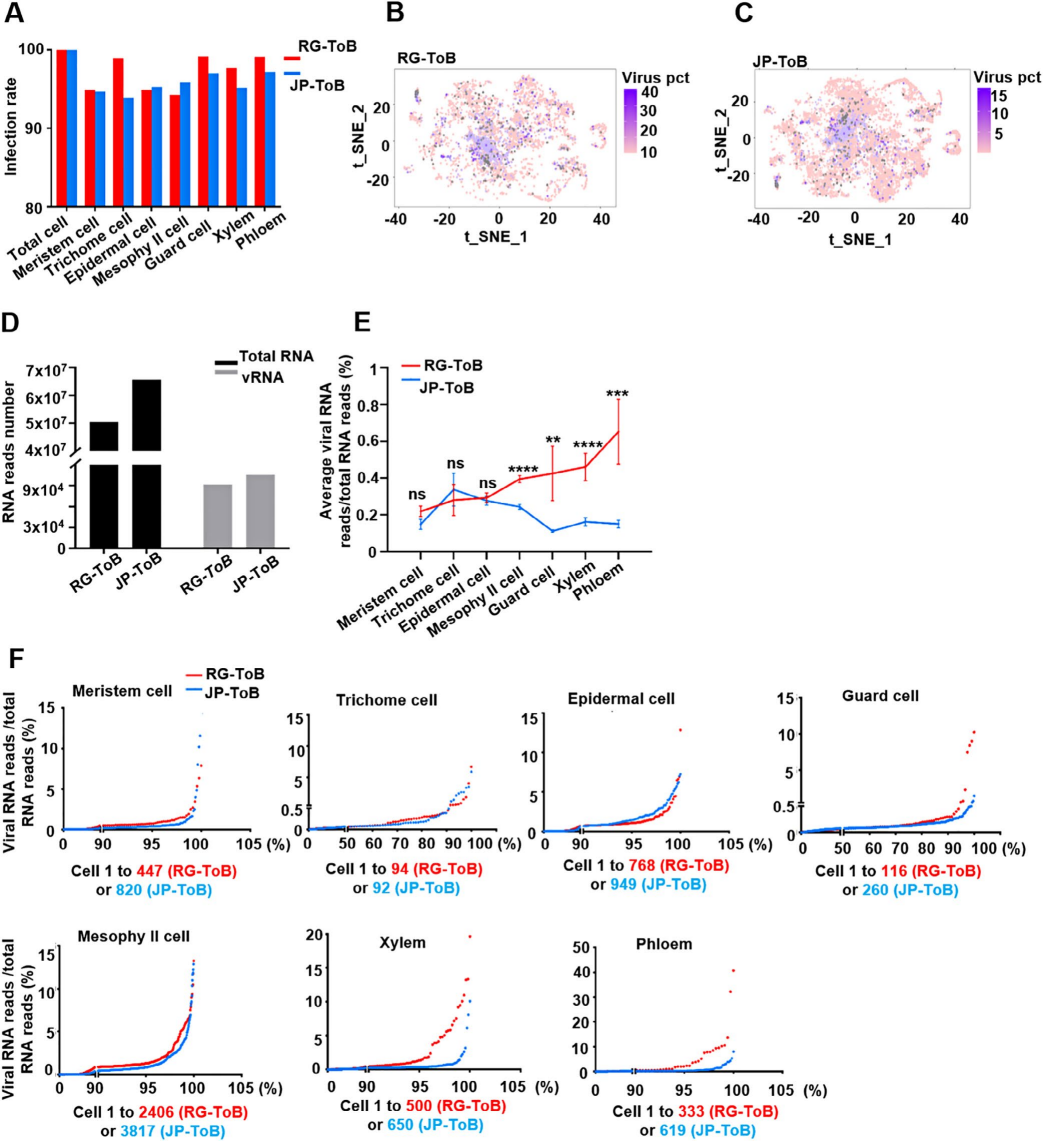
Cell type–specific modulation of ToBRFV accumulation in RG and JP tomato cultivars during early infection. (A) Infection rates of each cell type in ToBRFV‐infected RG and JP plants at 14 days post‐inoculation (dpi), based on the proportion of cells containing detectable levels of viral RNA. Over 90% of cells across all types were infected in both cultivars at this early stage. (B, C) t‐SNE projections present the distribution of viral loading in RG (B) and JP (C) plants. Grey dots represent non‐infected cells. (D) Total RNA and viral RNA abundance in RG‐ToB and JP‐ToB samples, represented by unique molecular identifiers (UMIs). JP‐ToB plants exhibited higher total and viral RNA counts due to a larger number of cells profiled. (E) Mean percentage of viral RNA reads relative to total RNA reads per cell type. ToBRFV accumulation was significantly lower in mesophyll, guard, xylem, and phloem cells of JP‐ToB plants compared to RG‐ToB plants, while no significant differences were observed in epidermal, trichome, or meristem cells. Data were analysed using Student's *t*‐test. **, *p* < 0.01; ***, *p* < 0.001; ****, *p* < 0.0001. (F) Distribution of viral RNA percentages across individual cells for each cell type. Cells were ranked in ascending order by viral RNA percentage to visualise cultivar‐specific trends. ToBRFV RNA levels were consistently lower in mesophyll, guard, xylem, and phloem cells of JP‐ToB plants. Red dots represent individual cells from RG‐ToB plants; blue dots represent cells from JP‐ToB plants.

The number of infected cells in each cell type was counted, and the percentage of infected cells relative to the total population was calculated. Over 90% of cells in each type were infected by ToBRFV in both cultivars at 14 dpi (Table S5 and Figure 3A). This early sampling suggests that JP does not significantly impede ToBRFV trafficking. T‐SNE projections were generated to provide an intuitive visualisation of viral load across cells in both JP (Figure 3B) and RG (Figure 3C) cultivars. To assess whether ToBRFV accumulates differently in cell types of RG and JP plants during early infection, both the absolute abundance (Figure 3D) and viral RNA proportion relative to total RNA (Figure 3E) were quantified. Total RNA and ToBRFV RNA read counts were higher in JP‐ToB plants than in RG‐ToB plants, likely due to the greater number of cells profiled in JP‐ToB samples (Figure 3D and Table S5). The proportion of viral RNA to total RNA was quantified for each cell, and the mean percentage was computed for each cell type across both cultivars. Comparative analysis showed that ToBRFV RNA accumulation was significantly reduced in mesophyll, guard, xylem, and phloem cells of JP‐ToB plants compared to RG‐ToB plants, while no significant differences were observed in meristem, trichome, or epidermal cells (Figure 3E). Notably, the highest proportion of viral RNA was observed in trichome cells of the JP (Figure 3E). However, the proportion of infected trichome cells in JP‐ToB leaves was the lowest among all cell types (Figure 3A), indicating trichome cells may help to limit ToBRFV spread in JP plants.

The mean percentage may be influenced by outlier cells exhibiting extreme values; to mitigate this potential bias, all cells of the same type were ordered ascendingly based on their viral RNA percentage to provide a comprehensive overview of viral distribution across individual cells, enabling direct comparison between JP‐ToB and RG‐ToB plants (Figure 3F). Consistent with the findings illustrated in Figure 3E, ToBRFV RNA accumulation was uniformly lower in mesophyll, guard, xylem, and phloem cells of JP‐ToB plants relative to RG‐ToB plants. No obvious differences were detected in other cell types, confirming the cultivar‐specific, cell type‐dependent viral RNA distribution pattern.

These findings suggest that ToBRFV accumulation was selectively regulated in the JP cultivar, leading to limited viral spread and reduced infection severity.

### 2.4 Cell‐Type Specific Gene Expression Changes in Tomato Leaves During ToBRFV Infection

**FIGURE 4:**
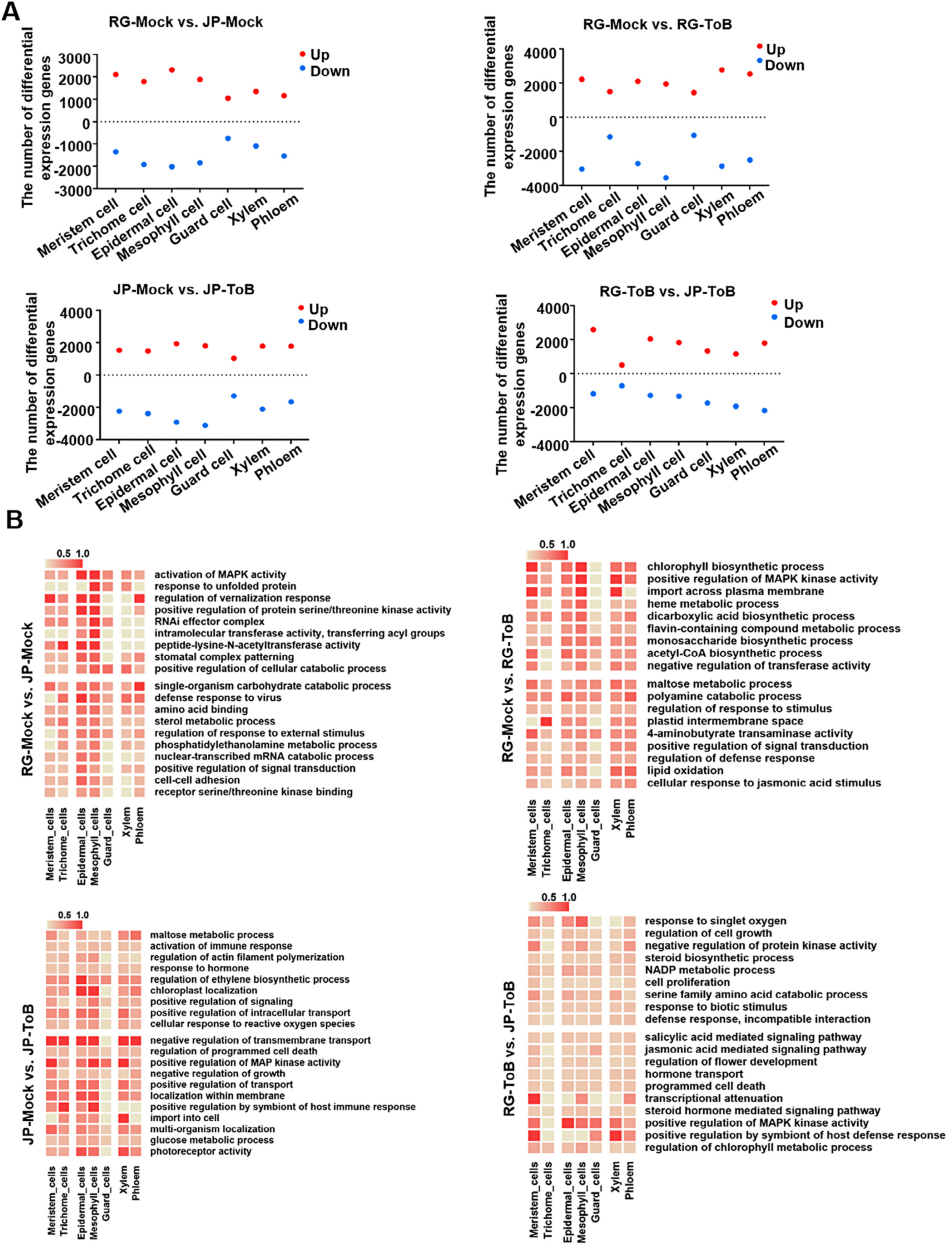
Cell type–specific transcriptomic responses to ToBRFV infection in RG and JP tomato cultivars. (A) Scatter plots depicting the number of DEGs identified within each cell type across four comparison groups: RG‐Mock vs. JP‐Mock, RG‐Mock vs. RG‐ToB, JP‐Mock vs. JP‐ToB, and RG‐ToB vs. JP‐ToB. (B) Heatmap illustrating GO term enrichment for DEGs in each cell type across the same four comparison groups. The colour intensity represents the enrichment factor of the genes, indicating the degree of functional overrepresentation.

To investigate transcriptomic alterations in different cell types following infection, gene expression profiles were compared between cells from ToBRFV‐inoculated and mock‐inoculated RG and JP plants. Differentially expressed genes (DEGs) were identified for each cell type using thresholds of |log2 FC| > 0.35 and *p* < 0.05. Firstly, DEGs between mock‐inoculated RG and JP plants were assessed. Epidermal cells had the most DEGs, with 4329 genes differentially expressed (2312 upregulated, 2017 downregulated), while guard cells had the fewest, with about 1793 DEGs (1043 upregulated, 750 downregulated) (Figure 4A). Among these, 143 upregulated and 121 downregulated DEGs were consistently observed across all seven cell types (Figure S3A). Gene Ontology (GO) pathway analysis revealed that these universally significant DEGs of all cell types were enriched in pathways including ‘activation of MAPK activity,’ ‘positive regulation of cellular catabolic process,’ ‘single‐organism carbohydrate catabolic process,’. Notably, DEGs in epidermal and mesophyll cells showed specific enrichment in the pathways related to ‘intramolecular transferase activity transferring acyl groups.’ Additionally, except for meristem cells, DEGs from the other six cell types were significantly enriched in pathways associated with the ‘defense response to viral infection’ and the ‘regulation of response to external stimulus’, suggesting that meristem cells may have distinct functional profiles compared to differentiated tissues (Figure 4B and Table S6). Comparison between JP‐ToB and JP‐Mock plants revealed that mesophyll cells had the most DEGs with 4922 genes (1811 upregulated, 3111 downregulated), followed by epidermal cells with 4860 DEGs (1942 upregulated, 2918 downregulated). Guard cells had the fewest, about 2330 DEGs (1039 upregulated, 1291 downregulated) (Figure 4A). A subset of DEGs was shared across multiple cell types, comprising 202 upregulated and 143 downregulated genes (Figure S3A). GO pathway analysis revealed that these significant DEGs associated with viral infection response were enriched in pathways such as ‘activation of immune response,’ ‘positive regulation of signaling,’ ‘positive regulation of MAPK kinase activity,’ and ‘regulation of programmed cell death’. Notably, DEGs in epidermal cells were significantly enriched in pathways such as the ‘regulation of ethylene biosynthetic process,’ while DEGs in both epidermal and mesophyll cells showed strong enrichment in the ‘chloroplast localization’ and ‘regulation of actin filament polymerization’ pathways. Moreover, DEGs from epidermal, mesophyll and xylem cells were markedly enriched in the pathway ‘photoreceptor activity’. Interestingly, DEGs from trichome and mesophyll cells were markedly enriched in the pathway ‘positive regulation by symbiont of host immune response’. In contrast, GO terms related to transport pathways, including ‘positive regulation of intracellular transport’ and ‘negative regulation of transmembrane transport’, were enriched across all cell types except guard cells. This pattern highlights distinct functional specialisations, with transport‐related processes widely active except in guard cells, which may utilise alternative regulatory mechanisms (Figure 4B and Table S7). In the comparison of RG‐ToB and RG‐Mock samples, xylem cells had the most DEGs with 5646 genes (2783 upregulated, 2863 downregulated), followed by mesophyll cells with 5502 DEGs (1960 upregulated, 3542 downregulated). Guard cells had the fewest, about 2507 DEGs (1447 upregulated, 1060 downregulated) (Figure 4A). Among these, 276 upregulated and 205 downregulated DEGs were consistently observed across all seven cell types (Figure S3A). GO pathway analysis revealed that DEGs associated with viral infection response in different cell types were significantly enriched in pathways including the ‘regulation of response to stimulus,’ ‘cellular response to jasmonic acid stimulus,’ and ‘regulation of defense response’. Additionally, DEGs showed significant enrichment in the pathways ‘positive regulation of MAPK kinase activity’ and ‘chlorophyll biosynthetic process’ in all cell types except guard cells. Interestingly, significant DEGs from trichome cells did not show enrichment in pathways like the ‘acetyl‐CoA biosynthetic process’ and ‘negative regulation of transferase activity,’ which were present in the other six cell types. This suggests that trichome cells may have distinct metabolic and regulatory profiles compared to the rest of the cell types analysed (Figure 4B and Table S8). DEGs were also identified between RG‐ToB and JP‐ToB samples. Notably, the highest number of DEGs was observed in phloem cells, totaling 3969 genes (1798 upregulated and 2171 downregulated) (Figure 4A). Among these, 100 upregulated and 83 downregulated DEGs were common across all seven cell types (Figure S3A). GO pathway analysis demonstrated that these shared DEGs were significantly enriched in pathways such as ‘steroid hormone‐mediated signaling pathway,’ ‘regulation of chlorophyll metabolic process,’ and ‘salicylic regulation of protein kinase activity.’ The DEGs of meristem, mesophyll and phloem cells were notably enriched in pathway ‘transcriptional attention’. Notably, the pathway of ‘positive regulation by symbiont of host defense response’ was significantly enriched in meristem, guard, xylem and phloem cells. Additionally, DEGs in mesophyll cells showed marked enrichment in ‘response to singlet oxygen’ (Figure 4B and Table S9). Additionally, KEGG pathway analysis of DEGs in ToBRFV‐ and mock‐inoculated RG/JP plants revealed significant enrichment in pathways related to ‘Ribosome,’ ‘Biosynthesis of amino acids,’ ‘Photosynthesis‐antenna proteins,’ and ‘Carbon metabolism’ (Figure S3B). These enrichments highlight both common and cell‐type‐specific regulatory mechanisms involved in hormonal signalling, metabolism, and host defence across different tissues.

### 2.5 Pseudotime Analysis of Signalling Pathways Involved in Tomato Plant Resistance to ToBRFV

**FIGURE 5:**
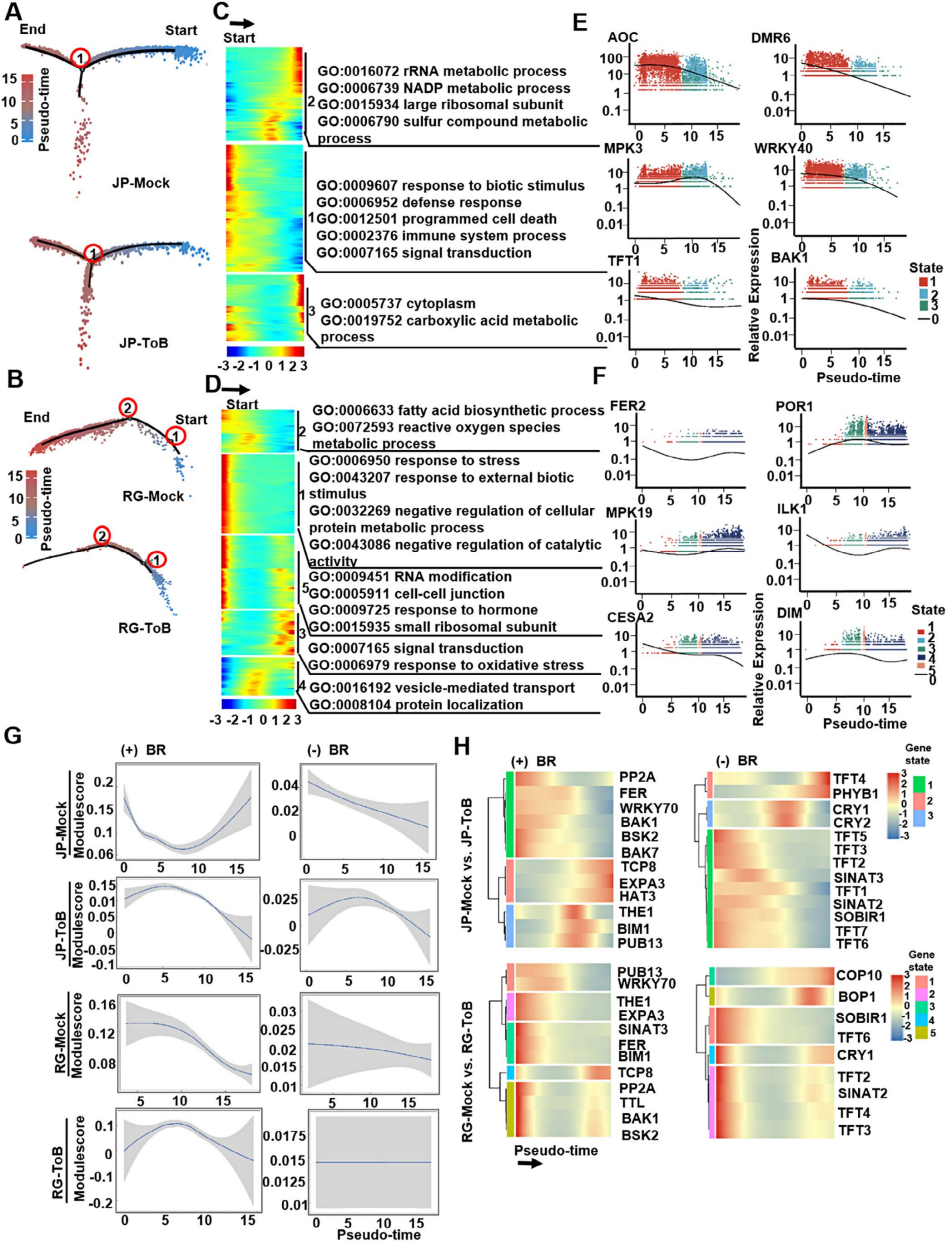
Pseudotime analysis of signalling pathways involved in tomato mesophyll cell responses to ToBRFV infection. (A, B) Developmental trajectories of mesophyll cells inferred using Monocle 2. Trajectory plots show pseudotime progression of individual mesophyll cells derived from JP‐Mock and JP‐ToB samples (A) and RG‐Mock and RG‐ToB samples (B). Each dot represents a single cell, coloured by its pseudotime value. A single branch point was detected in JP samples, whereas RG samples exhibited two branch points, indicating higher transcriptional heterogeneity upon ToBRFV infection. (C, D) Heatmaps displaying the expression of pseudotime‐dependent genes in JP‐ToB (C) and RG‐ToB (D) mesophyll cells, grouped by hierarchical clustering. Enriched GO terms for each cluster highlight activation of defence response, signal transduction, and reactive oxygen species metabolic processes along the developmental trajectory. (E, F) Expression dynamics of selected signal transduction‐related DEGs over pseudotime. Genes including *AOC*, *DMR6*, *MPK3*, *WRKY40*, *TFT1*, and *BAK1* were highly expressed during early pseudotime stages in JP‐ToB samples (E), whereas *FER2*, *POR1*, *MPK19*, *ILK1*, *CESA2*, and *DIM* were upregulated at later stages in RG‐ToB samples (F), suggesting distinct temporal gene regulation patterns between resistant and susceptible backgrounds. (G) Correlation between expression of BR pathway regulators and pseudotime. Line plots show regression trends of BR‐positive regulators (+) and BR‐negative regulators (−) across mesophyll cell pseudotime in JP‐Mock, JP‐ToB, RG‐Mock, and RG‐ToB samples. Notably, BR‐positive regulators such as *BAK1*, *BSKs*, *BIM1*, and *PP2A* declined over pseudotime in JP‐ToB cells, while BR‐negative regulators such as *TFTs*, *CRYs*, and *SINAT3* showed decreased expression in all samples except RG‐ToB. (H) Heatmaps illustrating the expression patterns of BR pathway DEGs with the most significant pseudotime‐associated changes. In JP‐ToB samples, many BR‐positive regulators exhibited a gradual or biphasic decline, while in RG‐ToB samples, expression trends were more variable, with several regulators showing biphasic patterns.

The mesophyll layer functions not only as the primary site of photosynthesis but also plays a critical role in shaping plant phenotypic responses to viral infection. To elucidate the developmental paths of individual tomato mesophyll cells during ToBRFV infection and identify key regulatory genes involved in their differentiation, Monocle 2 was employed to infer pseudotime paths. Gene expression matrices derived from mesophyll cells of JP‐ToB and JP‐Mock plants (Figure 5A), as well as from RG‐ToB and RG‐Mock plants (Figure 5B), were utilised to construct these developmental trajectories. The pseudotime trajectory for the JP‐Mock and JP‐ToB samples exhibited a single branch point (read circle), resulting in the classification of the cell population into three distinct states (Figure 5A and Figure S4A). In contrast, the RG‐Mock and RG‐ToB samples displayed a more complex trajectory with two branch points (read circles), dividing the cell population into five discrete states (Figure 5B and Figure S4A).

Investigation of pseudotime‐dependent genes revealed that pathways related to defence response, signal transduction, and reactive oxygen species metabolic processes were significantly enriched in response to ToBRFV infection in both JP (Figure 5C) and RG plants (Figure 5D). Additionally, six signal transduction‐related DEGs (*AOC*, *DMR6*, *MPK3*, *WRKY40*, *TFT1*, and *BAK1*) showed substantial fold changes over pseudotime and were highly expressed during the early stages of mesophyll cell development in the JP‐ToB (Figure 5E). In contrast, six different signal transduction‐related DEGs (*FER2*, *POR1*, *MPK19*, *ILK1*, *CESA2*, and *DIM*) were predominantly expressed in the late stages of mesophyll cell development in the RG‐ToB samples (Figure 5F).

Notably, many signal transduction‐related DEGs identified in both RG‐ToB and JP‐ToB samples are associated with the BR signalling pathway, including genes such as *WRKY40*, *MPK3*, *BAK1*, and *FER2*. This observation prompted an in‐depth investigation of BR‐related gene expression dynamics within mesophyll cells. Given that the tomato genome database is less comprehensive than that of Arabidopsis, a total of tomato homologues of 58 BR‐related genes from the Arabidopsis genome—comprising 34 positive and 24 negative regulators (Table S10)—were analysed for their expression patterns. Pseudotime analysis is a computational approach used in single‐cell RNA sequencing to infer the temporal order of cells along a dynamic biological process based on their gene expression profiles. By leveraging transcriptomic similarities, cells are arranged on a continuous trajectory that represents progression through states such as differentiation or development. This method assigns each cell a pseudotime value that reflects its relative position from early to late stages within the process, enabling reconstruction of cellular dynamics from snapshot single‐cell data without relying on actual time‐point measurements. Correlation analyses were performed between the expression levels of these positive and negative regulators in mesophyll cells and their respective pseudotime values to elucidate the relationship between BR signalling and the reduced accumulation of ToBRFV in JP plants. Key positive regulators, including *BSKs*, *BAK1*, *BIM1*, and *PP2A*, exhibited a gradual decline in expression along increasing pseudotime in mesophyll cells from JP‐ToB plants, whereas an inverse pattern was observed in JP‐Mock samples (Figure 5G). In contrast, no clear correlation was detected between positive regulator expression and pseudotime in RG‐ToB mesophyll cells, which, similarly to RG‐Mock plants, generally displayed a decreasing trend (Figure 5G). Key BR pathway negative regulator genes, such as *TFTs* (14–3‐3 genes), *CRYs*, and *SINAT3*, showed a gradual decrease in expression along increasing pseudotime values in mesophyll cells of JP‐Mock, JP‐ToB, and RG‐Mock plants. However, no significant changes were observed in RG‐ToB mesophyll cells (Figure 5G). The expression of DEGs in the BR pathway, including both positive and negative regulators, was analysed along pseudotime values in the RG‐Mock vs. RG‐ToB and JP‐Mock vs. JP‐ToB groups. In the JP‐Mock vs. JP‐ToB group, key positive regulator genes such as *BAK1*, *BSKs*, and *PP2A* showed a gradual decline in expression with increasing pseudotime. Conversely, other positive regulators like *THE1* and *BIM1* exhibited an initial increase in expression followed by a decrease as pseudotime progressed. Negative regulator genes (*TFTs*, *SINAT2*, *SOBIR1*) generally showed a downward trend in expression along pseudotime; however, *TFT4* and *PHYB1* were exceptions, displaying increased expression over the same period. In the RG‐Mock vs. RG‐ToB groups, most positive regulators, including *THE1*, *EXPA3*, *BIM1*, and *BOP1*, exhibited decreasing expression trends with advancing pseudotime. Yet, certain positive regulators (*TTL*, *BAK1*, *BSK2*) and negative regulators (*CRY1*, *TFT2*, *SINAT2*) presented biphasic expression patterns, with expression levels initially dropping and then rising later in pseudotime (Figure 5H). These findings suggest that positive regulators of the BR pathway may play an active role in the JP‐mediated defence response against ToBRFV infection.

Expression patterns of genes related to other hormone pathways were also analysed (Figure S4B). A strong correlation was found between the expression levels of ABA‐related positive regulators and pseudotime values in mesophyll cells of RG‐ToB plants, with these genes showing a progressive increase in expression as pseudotime advanced. In contrast, this correlation was not found in RG‐Mock plants. This indicates that ABA‐related positive regulators may play a role in RG plants during ToBRFV infection.

### 2.6 Involvement of BR‐Related Genes Against ToBRFV Infection in JP Cultivar

To investigate the expression patterns of hormone pathway‐related genes in response to ToBRFV infection in tomato mesophyll cells, GO pathway analysis was conducted on DEGs. Compared to RG‐Mock samples, the GO terms ‘jasmonic acid metabolic process’ and ‘salicylic acid‐mediated signaling pathway’ were enriched in JP samples in the absence of ToBRFV infection. These pathways were also predominantly activated by ToBRFV infection in mesophyll cells of both RG and JP plants.

The DEGs between mesophyll cells of RG‐ToB and JP‐ToB samples were significantly and specifically enriched in several signalling pathways, including the ‘jasmonic acid metabolic process,’ ‘steroid hormone‐mediated signaling pathway,’ and ‘ethylene metabolic process’ (Figure S5A). It is worth noting that BRs are plant steroid hormones. The role of ethylene in plant defence against viruses is complex and somewhat controversial. While ethylene is induced upon viral infection and can facilitate virus invasion (Zhao et al. 2017), it also contributes to resistance in certain interactions by cooperating with jasmonic acid (JA) and salicylic acid (SA) pathways (Yang et al. 2015).

BR signalling plays a key role in viral infection tolerance. Previous studies show that BR biosynthesis‐related gene expression is elevated in TMV‐infected *N. benthamiana* leaves, boosting resistance against TMV (Nakashita et al. 2003). To further explore hormone pathway dynamics, expression patterns of hormone‐related genes were analysed in tomato (Figure S5B), revealing profound alterations upon ToBRFV infection. Compared to mock‐infected JP plants, ToBRFV infection in JP plants caused significant upregulation of BR pathway genes such as *HERK1*, *CESA2*, *TCP8*, *DIM*, *THE1*, *EXPA3*, *PAR1*, and *MPK3*, while *DWF5* and *WRKY40* were markedly downregulated. A similar expression pattern was observed for most of these BR‐related genes in RG plants following infection, with the exception that *DWF5* was upregulated in RG‐ToB. In addition, JA‐related genes, including *JMT*, *J*RG*21*, and *AOC*, along with SA‐related genes such as *MLO6* and *DMR6*, were downregulated in JP‐ToB plants. Most of these JA‐ and SA‐related genes showed a comparable downregulation in RG‐ToB plants, except for *MLO6*, which was upregulated in RG after infection. Furthermore, ethylene pathway genes *CCR4* and *KRP1* were upregulated, while *ERF1* was downregulated in both JP and RG plants after ToBRFV infection. ABA‐related genes such as *ABR1* and *PED1* were downregulated in ToBRFV‐infected JP and RG plants, while *PP2CA* and *PUB9* were significantly upregulated. Cytokinin‐related genes such as *RVA1*, *LOG1*, *ARR1*, and *CKX5*, and the auxin pathway related genes such as *4CLL9*, RG*G2*, and *CIPK6* were downregulated in JP and RG plants following ToBRFV infection. Besides, compared to mock‐infected JP plants, ToBRFV infection in JP plants caused significant downregulation of gibberellin pathway genes, such as *LBD41*, *ERF1B*, and *GA2OX2*, while *SN2* was significantly upregulated. A similar expression pattern was observed for most of these gibberellin‐related genes in RG‐ToB plants relative to RG‐Mock plants, with the exception that *GA2OX2* was upregulated in RG plants following infection.

To investigate the role of BR regulators in ToBRFV infection, eight candidate genes were selected from the DEGs in the pseudotime trajectory and enriched pathways (Figure 5H and Figure S5B). The selected genes include delta(7)‐sterol reductase (*DWF5*), *DIM* (also known as DWARF1 or DIMINUTO/DWARF1), TCP domain protein 8 (*TCP8*), expansin A3 (*EXPA3*), cellulose synthase A2 (*CESA2*), mitogen‐activated protein kinase 3 (*MPK3*), hercules receptor kinase 1 (*HERK1*), and jasmonic acid carboxyl methyltransferase (*JMT*). These genes were categorised into four functional groups based on their roles within the BR signalling pathway.

**FIGURE 6:**
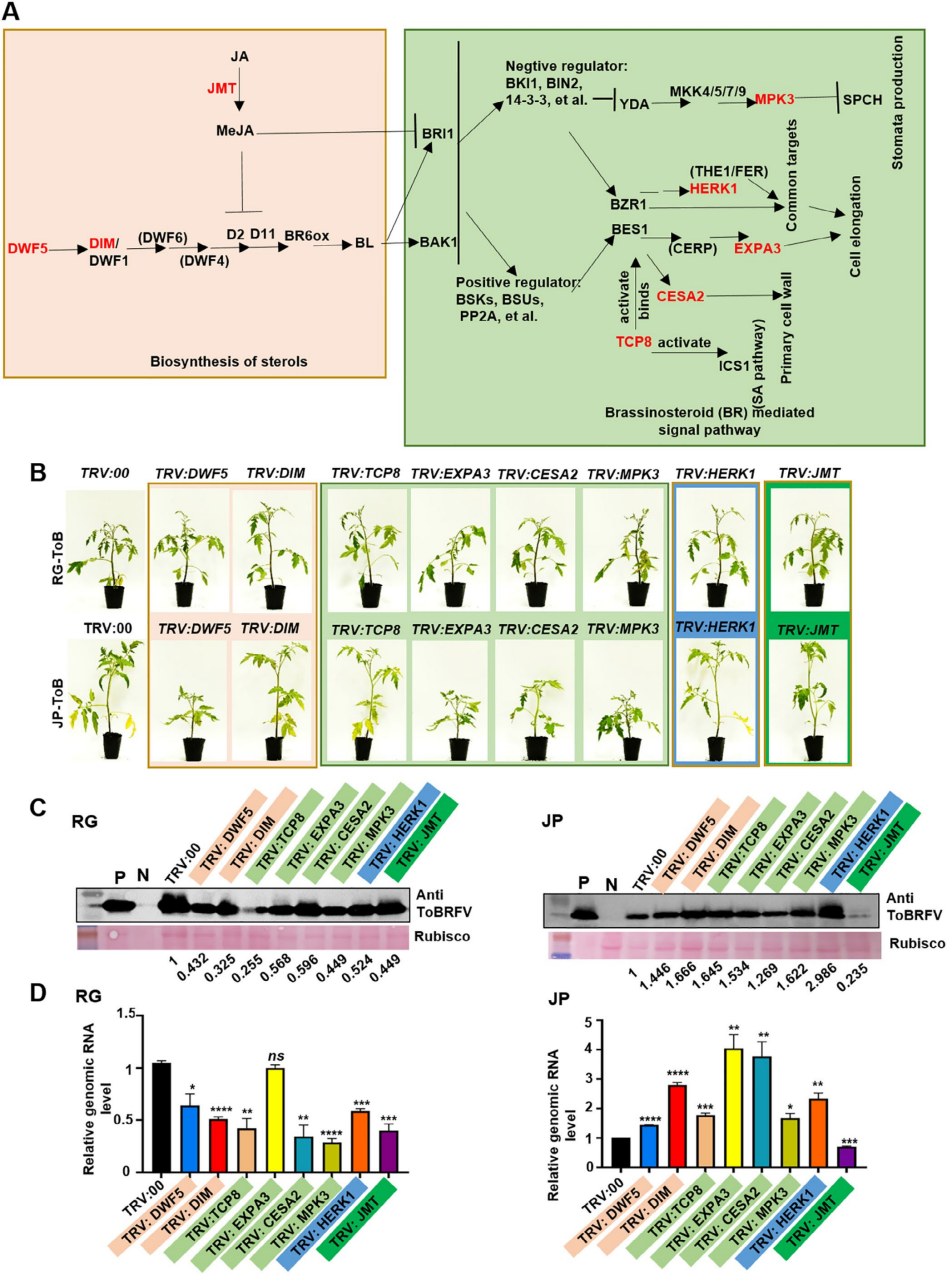
Functional roles of BR‐related genes during ToBRFV infection in two tomato cultivars. (A) Schematic model summarising the proposed roles of BR‐related genes in BR biosynthesis and signalling. *DWF5* and *DIM* are essential enzymes in BR biosynthesis. TCP8, *EXPA3*, *CESA2*, and *MPK3* act as positive regulators of BR signalling. *HERK1* operates in a pathway parallel to BR signalling, contributing to BR‐regulated cell elongation but not directly mediating BR responsiveness. In contrast, *JMT* acts as a suppressor of BR biosynthesis by producing methyl jasmonate, which negatively regulates BR pathways as part of hormonal crosstalk between growth and defence signalling. (B) Disease symptoms observed on systemic leaves at 12 days post‐inoculation (dpi) with ToBRFV in RG (upper) and JP (lower) tomato plants following VIGS‐mediated silencing of BR‐related genes (*DWF5*, *DIM*, *TCP8*, *EXPA3*, *CESA2*, *MPK3*, *HERK1*, and *JMT*). TRV:00‐inoculated plants served as negative controls. (C) Western blot detection of ToBRFV coat protein (CP) accumulation in systemic leaves of VIGS‐treated RG and JP plants at 12 dpi. (D) QRT‐PCR quantification of ToBRFV genomic RNA (RdRp) accumulation. Student's *t*‐test was applied for data comparisons. *, *p* < 0.05; **, *p* < 0.01; ***, *p* < 0.001; ****, *p* < 0.0001.

*DWF5* and *DIM* are essential enzymes in BR biosynthesis, with *DWF5* acting as a 7‐dehydrocholesterol reductase catalysing isofucosterol production, and *DIM* converting 24‐methylenecholesterol to campesterol, a key early step in BR and sterol synthesis (Klahre et al. 1998; Zolkiewicz et al. 2025). *TCP8*, *EXPA3*, *CESA2*, and *MPK3* are positive regulators of BR signalling. TCP8 enhances BR signalling by activating promoters of key transcriptional regulators *BZR1* and *BZR2*/*BES1* and interacting with these master regulators, with *tcp8* mutants showing altered BR responses (Spears et al. 2022). *EXPA3*, part of the *BES1*‐*CERP*‐*EXPA3* cascade, promotes cell elongation downstream of BR signalling (Zhu, Wang, et al. 2023), while *CESA2*, a cellulose synthase subunit regulated by *BES1*, supports cellulose synthesis needed for cell growth and can substitute for *CESA6* in BR‐induced growth (Xie et al. 2011). *HERK1*, a *CrRLK1L* family receptor kinase, acts in a pathway supportive or parallel to BR signalling (Guo et al. 2009), regulated by *BES1* but functioning largely independently to regulate growth, as *HERK1* mutants retain normal BR responsiveness (Guo et al. 2009). Conversely, *JMT* suppresses BR biosynthesis by converting JA to methyl jasmonate, which negatively regulates both JA signalling and BR biosynthesis, illustrating complex hormonal crosstalk balancing growth and defence (Gan et al. 2015; Wang et al. 2021) (Figure 6A). Knockdown of *DWF5*, *DIM*, *TCP8*, *EXPA3*, CESA2, *MPK3*, *HERK1*, and *JMT* was performed via VIGS in RG and JP tomato plants, with TRV:00‐inoculated plants as controls. VIGS vectors were constructed, and TRV:PDS plants showed leaf bleaching at 7 dpi, confirming VIGS efficiency (Figure S6A). All target gene expression levels were reduced by ~50% compared to TRV:00 (Figure S6B), with no visible phenotypic abnormalities in either background (Figure S6C). These plants were then agro‐inoculated with ToBRFV. At 12 dpi, silencing of all genes except *EXPA3* in RG plants led to reduced symptoms, including milder leaf curling. *EXPA3*‐silenced plants showed dwarfism compared to TRV:00 controls (Figure 6B). Detailed disease phenotypes are further illustrated in Figure S6D. ToBRFV CP accumulated at lower levels in plants silenced for each candidate gene compared to TRV:00 controls (Figure 6C). ToBRFV genomic RNA (RdRp) levels were reduced by ~50% in plants silenced for all genes except *EXPA3*, with no significant difference between *EXPA3*‐silenced and TRV:00 control plants (Figure 6D). These findings suggest that BR‐related genes promote ToBRFV infection in RG tomato plants.

At 12 dpi, systemic lower leaves of JP plants silenced for *DWF5*, *EXPA3*, *CESA2*, and *MPK3* showed more severe symptoms, including mosaic patterns and dwarfism, compared to TRV:00 controls (Figure 6B). ToBRFV CP accumulation was higher in plants silenced for all candidate genes except JMT, where it was significantly reduced (Figure 6C). Similarly, ToBRFV genomic RNA levels were elevated in all silenced groups except *JMT* (Figure 6D).

To quantify endogenous BR levels in RG and JP cultivars following ToBRFV infection, we performed high‐performance liquid chromatography‐mass spectrometry (HPLC‐MS) analysis. Standard curves were generated using known BR concentrations under optimised conditions (Figure S7A), yielding a consistent retention time of 3.26 min and characteristic ion profiles (Figure S7B,C). The calibration curves showed excellent linearity (*R* ^*2*^ = 0.9952) with detection limits ranging from 0.48 to 1000 ng/mL (Figure S7D), confirming the accuracy and sensitivity of the method for BR quantification. Following ToBRFV infection, BR levels significantly decreased in RG plants but increased in JP‐ToB plants (Figure S7E). At 12 dpi, systemic leaves from BR‐related gene–silenced plants were analysed by HPLC‐MS. Most silenced RG plants showed elevated BR levels compared with the TRV:00 control, except for *DIM*, *EXPA3* and *MPK3* (Figure S7F), whereas all silenced JP plants exhibited reduced BR accumulation, although the differences were not statistically significant in some cases (Figure S7G). These contrasting responses indicate that BR signalling functions differently in the two cultivars during ToBRFV infection. Silencing of *JMT* did not significantly affect BR levels in either cultivar. Notably, BR levels were higher in RG‐Mock than in JP‐Mock plants, a discrepancy that warrants further investigation.

### 2.7 Exogenous Brassinolide Inhibited ToBRFV Infection in JP Cultivar but Plays an Opposite Role in RG Cultivar

**FIGURE 7:**
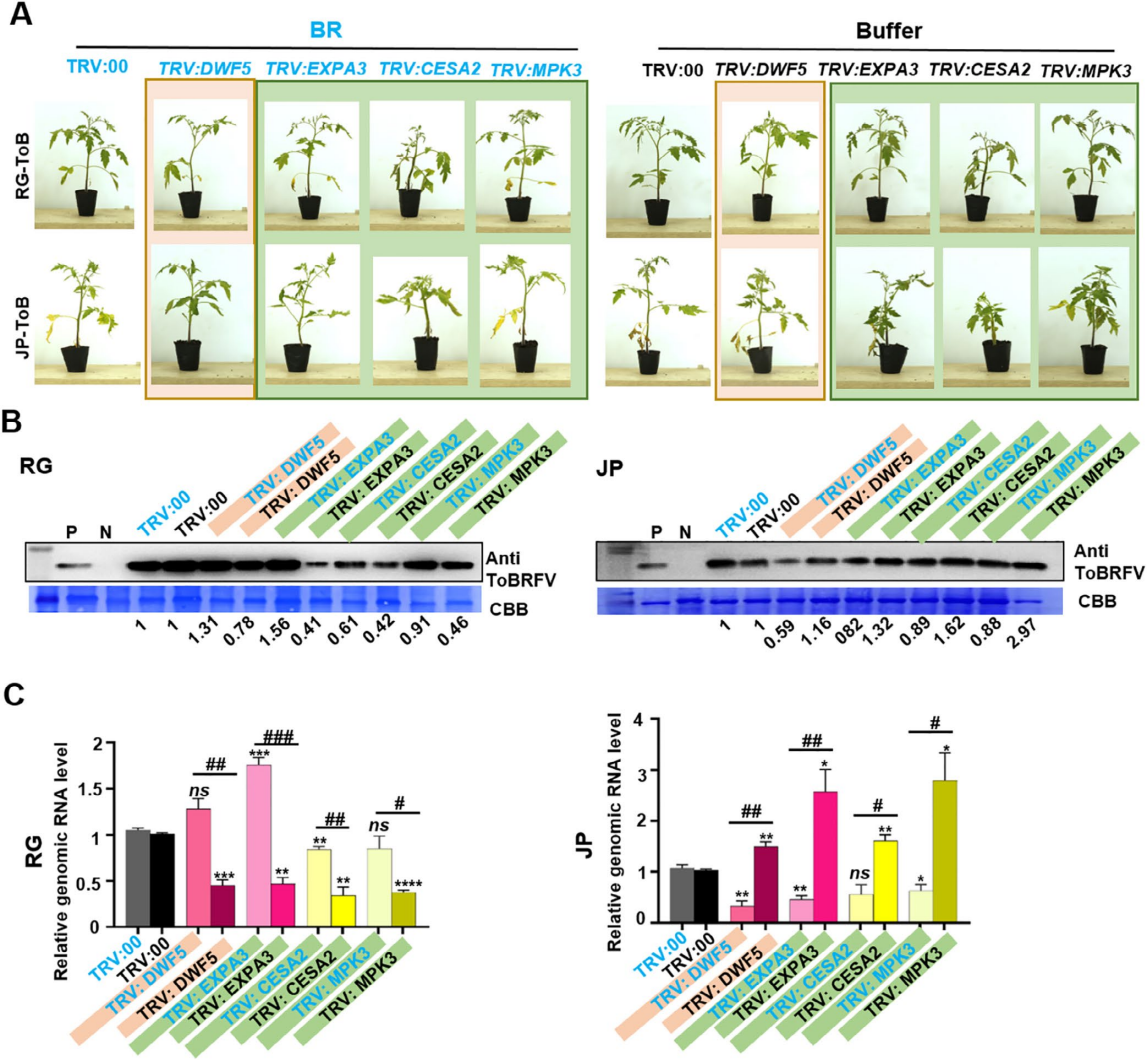
Role of EBL in ToBRFV infection in two tomato cultivars. (A) Disease symptoms observed on systemic leaves of RG (upper panel) and JP (lower panel) tomato plants at 12 days post‐inoculation (dpi) with ToBRFV, following VIGS‐mediated silencing of BR‐related genes (*DWF5*, *EXPA3*, *CESA2*, and *MPK3*). TRV:00‐inoculated plants served as negative controls. (B) Immunoblot detection of ToBRFV coat protein (CP) accumulation in systemic leaves of VIGS‐treated RG and JP plants at 12 dpi. (C) QRT‐PCR quantification of ToBRFV genomic RNA (RdRp) accumulation. Student's *t*‐test was applied for data comparisons. *, #, *p* < 0.05; **, ##, *p* < 0.01; ***, ###, *p* < 0.001; ****, *p* < 0.0001. *Indicate comparisons between gene‐silenced plants and TRV:00 controls treated with EBL; # indicate comparisons between gene‐silenced plants treated with EBL and buffer.

Exogenous 24‐epibrassinolide (EBL), an active brassinolide, was used to further confirm the role of BR in ToBRFV infection. Four candidate genes (*DWF5*, *EXPA3*, *CESA2*, and *MPK3*) were selected for further analysis based on their association with dwarfism observed 12 days after ToBRFV inoculation (Figure 6B). VIGS‐mediated gene silencing was performed as previously described (Figure 6B). At 7 dpi, TRV:PDS plants showed the characteristic leaf bleaching, confirming the efficiency of VIGS (Figure S8A). The expression levels of *DWF5*, *EXPA3*, *CESA2*, and *MPK3* were reduced by approximately 50% compared to TRV:00 controls (Figure S8B), without causing any obvious phenotypic abnormalities in either cultivar (Figure S8C). Detailed disease phenotypes are further illustrated in Figure S8D. At this time point, the plants were agro‐inoculated with ToBRFV. Exogenous application of EBL was performed 24 h after agroinfiltration, once per day for 12 days, just prior to sampling and photography. Compared to RG plants without exogenous EBL treatment, disease symptoms were significantly more pronounced in each of the four gene‐silencing groups treated with EBL (Figure 7A), which corresponded with increased levels of CP protein (Figure 7B) and genomic RNA (Figure 7C). In contrast, in JP plants, EBL application alleviated disease symptoms (Figure 7A) and reduced the levels of both CP protein (Figure 7B) and genomic RNA (Figure 7C).

### 2.8 Genome‐Wide Variations Were Observed Between JP and RG Cultivars Beyond *Tm‐2^2^* and *tm‐2* Alleles

The genomes of RG and JP were sequenced with an average coverage depth exceeding 17× across all chromosomes (Figure S9A). To further investigate the genetic basis underlying the differing responses of the RG and JP tomato cultivars to ToBRFV infection, genome‐wide variations were analysed by aligning their genomes to the *Solanum lycopersicum* ( *S. lycopersicum* ) reference genome (SL3.0, Ensembl). Analysis of single nucleotide polymorphism (SNP) revealed that, in RG, 81.17% (258649) of SNPs were located in intergenic regions, while 18.83% (60362) were within genic regions (Figure S9B). Among the SNPs within genic regions, 9011 were found in coding regions, comprising 3131 synonymous and 5660 non‐synonymous SNPs. In JP, 82.44% (667688) of SNPs were intergenic, and 17.56% (143235) were genic, including 19 769 in coding regions (7489 synonymous and 11 897 non‐synonymous). Further analysis categorised SNPs by heterozygosity. In RG, homozygous SNPs comprised 62.30% (198732) and heterozygous SNPs 37.70% (120279). Conversely, JP exhibited 34.57% (280376) homozygous and 65.43% (530547) heterozygous SNPs.

Comprehensive genomic analysis identified 75 945 and 112 170 insertions or deletions (InDels) in RG and JP, respectively, with 636 and 1032 located in coding regions (CDs). In RG, insertions and deletions within coding regions were nearly balanced (50.00% vs. 48.43%), with 64.21% homozygous and 35.79% heterozygous mutations. JP showed a similar pattern, with insertions slightly more frequent than deletions (51.94% vs. 46.13%) and a higher proportion of heterozygous mutations (60.71%). InDels showed a relatively higher proportion within genic regions, accounting for 34.75% (26304) in RG and 31.96% (35726) in JP (Figure S9C). Of these, 636 InDels in RG and 1032 in JP were located in coding regions. Additionally, the chromosomal distribution of variants differed between the two cultivars, with JP showing a higher overall variant density (Figure S9D).

Copy number variation (CNV) analysis revealed 17 491 and 17 603 CNVs in RG and JP, respectively, with deletions far outnumbering duplications in both cultivars (15 962 vs. 1529 in RG; 15 781 vs. 1822 in JP), indicating that deletions represent the dominant type of structural variation.

Variant genes, the genes were found with SNPs and/or InDels, were identified. RG contained 3572 genes with sequence variations, including 3312 genes harbouring SNPs and 576 with InDels, whereas JP had 5699 variant genes, comprising 5433 with SNPs and 899 with InDels (Figure S10A), indicating significant functional divergence between the two cultivars. GO enrichment analysis categorised these genes into biological process, cellular component, and molecular function. RG showed enrichment in 13 cellular components, 8 molecular functions, and 9 biological processes (Figure S10B), whereas JP displayed enrichment in 7 cellular components, 12 molecular functions, and 11 biological processes (Figure S10C). Commonly enriched GO terms across both cultivars included photosynthesis, cellular process, and metabolic process in the biological process category; plastid, photosynthetic membrane, and thylakoid in the cellular component category; and catalytic activity and binding in molecular function, reflecting their potential roles in the distinct physiological responses to ToBRFV.

## 3 Discussion

Our study sheds light on the cellular dynamics of ToBRFV infection and the defence mechanisms in two tomato cultivars with different genetic backgrounds. We found that ToBRFV infection differentially influenced cell differentiation in the leaves of JP and RG tomato cultivars. While viral entry or movement was not fully prevented in JP plants, viral replication was selectively limited in specific cell types. We also identified distinct signalling pathways activated in response to ToBRFV in the two cultivars. Notably, the BR signalling pathway showed antiviral effects only in the JP cultivar, providing new insights into cultivar‐dependent plant‐virus interactions and highlighting potential targets for improving crop resistance.

Several studies have employed scRNA‐seq to investigate the cell‐type‐specific responses of host plants to various plant viruses, including tomato chlorosis virus (Yue et al. 2024), soybean mosaic virus (Song et al. 2024), and sugarcane mosaic virus (Chen et al. 2025). These studies offer valuable insights into plant‐virus interactions at the cellular level. However, cultivar‐specific responses were not addressed in these investigations. Given the genetic diversity among cultivars, differential interactions with viruses are likely, and conclusions drawn from studies using a single cultivar may not be broadly applicable to other cultivars. In this study, ToBRFV infection caused distinct alterations in leaf cell‐type composition between JP and RG cultivars, likely reflecting cultivar‐specific changes in signalling pathways across different cell types (Figure 4), such as hormone regulation, RNA silencing, and metabolism that can affect cell proliferation and differentiation (Kutsher et al. 2021; Laliberté and Sanfaçon 2010). These alterations may also be associated with the differential accumulation of ToBRFV in the two cultivars. Although the viral load was distributed across all cell types in both JP and RG plants, with similarly high early infection rates (over 90%), JP plants exhibited lower viral RNA accumulation in mesophyll, guard, xylem, and phloem cells compared with RG plants (Figure 3). This finding indicates that, in JP plants, viral entry or movement was not completely blocked at early stages but viral replication or accumulation was selectively restricted in key cell types critical for systemic infection. In contrast, viral RNA levels in trichome, epidermal, and meristem cells were less affected in JP cultivars. The reduced infection in trichomes from JP plants suggests they play a crucial role in limiting ToBRFV spread or triggering defence signalling.

The high viral load likely causes extensive cellular damage in highly susceptible RG plants. Microscopic observations show a strong correlation between elevated ToBRFV accumulation and widespread damage in key tissues, including mesophyll, guard, xylem, and phloem cells, essential for photosynthesis, gas exchange, and systemic transport. This significant viral presence disrupts cellular integrity, causing mesophyll cell rupture, stomatal abnormalities, and disorganised epidermal structure, which enhances viral replication and movement. In contrast, JP plants show reduced viral RNA levels in these cells, preserving cellular structure and function. The genetic differences between the JP and RG cultivars may contribute significantly to this variation in response. JP plants are characterised by their partial tolerance to ToBRFV, mediated by a unique genetic profile that includes the *Tm‐2* ^*2*^ gene, which encodes a CC‐NBS‐LRR protein (Lanfermeijer et al. 2005). By recognising the viral MP, this gene confers strong and durable resistance, characterised by the absence of visible lesions and effective suppression of viral symptoms without causing tissue necrosis (Zhang et al. 2013; Kobayashi et al. 2011; Lanfermeijer et al. 2005; Meshi et al. 1989; Weber and Pfitzner 1998). In this study, ToBRFV infection of JP plants leads to localised necrotic lesions, representing a controlled HR or programmed cell death, which acts as a barrier to restrict viral spread. Unlike TMV and ToMV, ToBRFV triggers an HR in JP plants, reflecting a different interaction dynamic and possibly less effective suppression of viral proliferation. Dehydration and disruption are confined to these lesions, sparing surrounding tissue and helping maintain plant growth despite infection. These cellular responses highlight the differential defence strategy of JP, where viral replication is suppressed in critical cells, while localised cell death limits infection. The necrotic lesions represent a balance between resistance and minimising damage, a hallmark of durable R gene‐mediated resistance, demonstrating how cell‐type‐specific antiviral defences were orchestrated in the JP cultivar to mitigate ToBRFV‐induced damage (Giolai and Laine 2024; Huot et al. 2014).

Cell‐type specific responses likely influence disease symptom manifestation. ToBRFV induces stomatal opening in RG plants compared to controls (Figure 1E). Phytohormones like ABA, BRs, and JA regulate stomatal dynamics under varying conditions (Burgess and Huang 2016; Haubrick and Assmann 2006). scRNA‐seq analysis revealed activation of pathways related to ‘cellular response to jasmonic acid stimulus’ in ToBRFV‐infected RG cells (Figure 4B). Microscopic observations revealed mesophyll cell rupture in both RG and JP plants infected with ToBRFV (Figure 1F). Pathogen infections are known to activate ROS‐producing enzymes such as NADPH oxidases and cell wall peroxidases, leading to the accumulation of ROS (Pogány et al. 2006). Excess ROS can trigger lipid peroxidation, membrane rupture, and cell death (Banerjee and Roychoudhury 2022; Ye et al. 2021). Transcriptomic analysis showed that DEGs in RG mesophyll cells were enriched in ‘response to singlet oxygen,’ while JP cells showed enrichment in ‘cellular response to reactive oxygen species’ (Figure 4B). Pseudotime analysis further revealed enrichment of ‘NADP metabolic process’ and ‘programmed cell death’ in JP mesophyll cells (Figure 5C), and ‘ROS metabolic process’ and ‘response to oxidative stress’ in RG mesophyll cells (Figure 5D). Notably, ‘signal transduction’ pathways were activated in both cultivars upon ToBRFV infection (Figure 5C,D). Previous studies suggest a synergistic role of BR and ABA in promoting ROS production and oxidative stress responses (Zhou et al. 2014). Together, these findings indicate that ToBRFV infection induces excessive ROS accumulation, leading to mesophyll cell membrane rupture in both JP and RG plants. Palisade mesophyll cells in ToBRFV‐inoculated RG and JP plants exhibited irregular, more circular shapes compared to those in mock‐inoculated plants (Figure 1F). Similar alterations were also observed in the morphology of lower epidermal cells of JP‐ToB plants (Figure 1F). Systemic leaves of RG‐ToB developed characteristic wiry and curled symptoms (Figure 1A). Previous studies reported that in tomato wiry leaf abaxial patches, palisade mesophyll cells were replaced by spongy mesophyll cells (Kim et al. 2003; Yifhar et al. 2012), suggesting that the irregular and circular palisade mesophyll cell shapes in RG‐ToB plants may be associated with the leaf‐wiry phenotype. However, wiry and curled symptoms were absent in JP‐ToB plants. The morphogenetic pattern of plant cells is regulated by cytoskeletal organisation in various cell types, including leaf mesophyll and epidermis. In maize, mesophyll cell morphogenesis results from coordinated local differentiation of the cell wall matrix and microtubule‐dependent alignment of cellulose microfibrils, enabling mesophyll cells to adopt their characteristic shape (Giannoutsou et al. 2013; Sotiriou et al. 2016). Additionally, expression of the blue‐light photoreceptor phototropin 2 in mesophyll cells of Arabidopsis promotes the development of cylindrical palisade cells in a tissue‐autonomous manner (Kozuka et al. 2011). scRNA‐seq analysis revealed that DEGs in mesophyll cells of JP‐ToB plants were significantly enriched for functions related to ‘regulation of actin filament polymerization’ and ‘photoreceptor activity’ (Figure 4B). These findings suggest that the morphological changes observed in the palisade mesophyll and epidermal cells of JP‐ToB plants may be driven by modulation of cytoskeletal dynamics or light signalling pathways.

A key discovery of this work is the critical role of the BR signalling pathway in ToBRFV infection. Transcriptomic and pseudotime trajectory analyses revealed differential expression dynamics of BR pathway components in mesophyll cells of JP plants during cell differentiation. Positive BR regulators, including *BAK1*, *BSKs*, *BIM1*, and *PP2A*, were downregulated along cell developmental pseudotime in infected JP plants, a pattern absent in RG plants. Negative regulators also showed distinct expression trends. These data suggest that BR signalling is modulated in JP plants to establish defence in susceptible cell types. Functional validation via VIGS revealed a cultivar‐dependent role of BR pathway genes. In RG plants, silencing BR biosynthesis (*DWF5*, *DIM*) and signalling (*TCP8*, *CESA2*, *MPK3*, *HERK1*) genes reduced ToBRFV symptoms and viral loads, indicating their role as susceptibility factors. In contrast, silencing these genes in JP plants increased symptom severity and viral accumulation. Similar expression patterns of eight BR‐related genes in both cultivars suggest a shared initial response to viral infection (Figure S6B). The downstream functional outcomes differ between the two cultivars, likely due to their distinct genetic backgrounds. Silencing *JMT*, a suppressor of BR biosynthesis and JA signalling, reduced ToBRFV accumulation in both cultivars. Exogenous BR application suppressed ToBRFV in JP plants but enhanced infection in RG plants. This context‐dependent dual role of BR signalling underscores the complex crosstalk between the BR and JA pathways, as well as the influence of cultivar‐specific factors. These findings align with evidence that BR signalling is a nuanced modulator of plant immunity, not simply a promoter or suppressor. The BR receptor kinase BAK1 plays a key role by interacting with immune receptors and facilitating signalling crosstalk involving MAPK cascades and ROS production (Ortiz‐Morea et al. 2020; Xiong et al. 2022), coordinating growth‐defence trade‐offs essential for resistance without high fitness costs (Ortiz‐Morea et al. 2020).

We speculate that the reduced viral accumulation and activated BR signalling in JP are at least partially influenced by the presence of *Tm‐2* ^*2*^, as although ToBRFV can overcome *Tm‐2* ^*2*^ via its MP, this is associated with a fitness cost (Hak and Spiegelman 2021). Future studies may examine the interactions among viral components (CP and MP), *Tm‐2* ^*2*^, and BR signalling regulators to clarify BR‐mediated antiviral mechanisms. However, our whole‐genome resequencing revealed that the genetic background of JP differs substantially from RG beyond the *Tm‐2* ^*2*^ and *tm‐2* alleles. Genetic background can strongly influence gene function. For instance, the expression of rice disease resistance gene *Xa3*/*Xa26* is regulated differently across genetic backgrounds and developmental stages (Cao et al. 2007). Furthermore, the effectiveness of the *Xa21* gene in controlling *Xanthomonas axonopodis pv. citri* also differs among sweet orange cultivars (Mendes et al. 2010). Beyond *Tm‐2* ^*2*^, other genetic factors in JP may also directly activate BR signalling or interact with viral factors to indirectly stimulate BR pathways. Further validation using RG and transgenic RG lines expressing *Tm‐2* ^*2*^ is needed to assess whether *Tm‐2* ^*2*^ plays a dominant role in this process.

## 4 Conclusion

This study demonstrates that the JP cultivar exhibits partial tolerance to ToBRFV, characterised by cell‐type‐specific reductions in viral accumulation and coordinated cellular responses involving BR signalling. The cultivar‐specific effects observed upon BR gene silencing reveal that BR pathway components act as susceptibility factors in RG plants, while functioning as critical mediators of antiviral defence in JP plants. These findings provide new insights into the molecular and cellular mechanisms underlying background‐specific tolerance and highlight the potential of manipulating hormone signalling pathways, such as BR, to enhance durable viral management in tomato and other crops.

## 5 Materials and Methods

The detailed description of the experimental materials and methods can be found in the Materials S1.

## Author Contributions

J.W. designed the experiments and supervised the research. Y.Z., S.B., Y.N., and L.W. performed experiments. J.L., J.X., and J.P. analysed data. Y.Z. wrote the manuscript. J.W. and F.Y. revised the manuscript.

## Funding

This study was supported by the National Natural Science Foundation of China (32470149, 32272483) to Jian Wu.

## Conflicts of Interest

The authors declare no conflicts of interest.

## Supporting information


**Data S1:** Supporting information.

**Figure S1:** Quality control of single‐cell sequencing data and expression level quantification.
**Figure S2:** Expression patterns of potential novel marker genes in tomato leaf cells.
**Figure S3:** DEGs and KEGG pathway enrichment in mock‐ and ToBRFV‐infected samples.
**Figure S4:** Differentiation trajectory of mesophyll cells.
**Figure S5:** Hormone signalling dynamics in tomato mesophyll cells during JP ‐mediated defence against ToBRFV infection.
**Figure S6:** Phenotypic characterisation of BR‐related gene‐silenced tomato plants.
**Figure S7:** The changes of endogenous BRs levels in tomato leaves upon ToBRFV infection in two tomato cultivars.
**Figure S8:** Phenotypic characterisation of BR‐related gene‐silenced tomato plants treated with EBL.
**Figure S9:** Identification and functional annotation of SNPs and InDels in two tomato cultivars.
**Figure S10:** Characterisation of genome‐wide genetic variations in two tomato cultivars.

**Table S1:** Detailed information of scRNA‐seq data of each group.

**Table S2:** Cell counts for each cluster in each group.

**Table S3:** Expression of maker genes.

**Table S4:** Cell numbers of each cell type in each group.

**Table S5:** Total and viral RNA read counts and infected cell percentages.

**Table S6:** GO annotation of DEGs and rich factors of GO pathways in each cell type: comparison between RG‐Mock and JP‐Mock groups.

**Table S7:** GO annotation of DEGs and rich factors of GO pathways in each cell type: comparison between JP‐Mock and JP‐ToB groups.

**Table S8:** GO annotation of DEGs and rich factors of GO pathways in each cell type: comparison between RG‐Mock and RG‐ToB groups.

**Table S9:** GO annotation of DEGs and rich factors of GO pathways in each cell type: comparison between RG‐ToB vs. JP‐ToB groups.

**Table S10:** List of genes annotated as ‘positive regulators of the BR signaling pathway’ and ‘negative regulators of the BR signaling pathway’ according to the TAIR database.

**Table S11:** Primer sequences.

## Data Availability

The raw scRNA‐seq data have been deposited in NCBI GEO under accession number GSE300843. The raw genome resequencing data have been deposited in NCBI SRA under accession number PRJNA1356722.
