# Supplementary material for: Dissecting the Cell‐Type‐Specific Response to an Emerging Tobamovirus in Tomato Reveals Cultivar‐Dependent Involvement of Brassinosteroid Signalling
Source: Plant Biotechnol J. 2026 Jan 21;24(5):3106–24. doi: 10.1111/pbi.70559 (PMC13110166; doi:10.1111/pbi.70559)
Supplement: Supplementary file 1 — Data S1: Supporting information. [file PBI-24-3106-s011.docx]

**Dissecting the cell-type-specific response to an emerging tobamovirus in tomato reveals cultivar-dependent involvement of brassinosteroid signaling**

Yuhong Zhang, Shan Bu, Yuxin Nie, Luyou Wang, Jiayi Liu, Junchen Xu, Jiejun Peng, Fei Yan and Jian Wu

State Key Laboratory for Managing Biotic and Chemical Threats to the Quality and Safety of Agro-products, Key Laboratory of Biotechnology in Plant Protection of MARA, Key Laboratory of Green Plant Protection of Zhejiang Province, Institute of Plant Virology, Ningbo University, Ningbo 315211, China

Correspondence: Fei Yan ([yanfei@nbu.edu.cn](mailto:yanfei@nbu.edu.cn)) and Jian Wu (wujian@nbu.edu.cn )

**Materials and Methods**

**Plants and agroinfiltration**

Tomato plants homozygous for JP (cv. “Jinpeng No. 1”) or RG (cv. “Rutgers”) were grown at 25°C with a 16-hour light/8-hour dark cycle. Jinpeng seeds were sourced from Xi'an Jinpeng Seedling Company, while Rutgers was grown in-house. Agrobacterium-mediated infection was performed at the two-cotyledon stage, and TRV-mediated gene silencing at the 2-leaf stage. Agrobacterium cultures with viral clones were infiltrated into cotyledon leaves at OD600 of 0.5. Systemic leaf samples were collected at 7, 12, and 14 dpi for qRT-PCR and western blot analysis.

**Vector construction and virus-induced gene silencing in tomato**

The full-length CDS of eight BR-related tomato genes (*DWF5*, *DIM*, *TCP8*, *EXPA3*, *CESA2*, *MPK3*, *HERK1*, *JMT*) were PCR-amplified from tomato cDNA using KOD FX DNA Polymerase. Partial gene fragments (272–299 nucleotides) were cloned into pTRV2 to create gene silencing vectors. For silencing, TRV vectors (TRV: target gene) were mixed 1:1 with pTRV1 and agroinfiltrated into 2-leaf stage tomato leaves. An empty vector (pTRV2:00) with pTRV1 served as a negative control. Seven days post-infiltration, silenced plants were used for further analyses, including ToBRFV inoculation. Primer sequences are listed in Table S11.

**Protein preparation and western blot**

To assess ToBRFV CP accumulation in systemic leaves, total protein extracts were prepared as previously described (Han et al., 2023). Proteins (50 ng per lane) were separated by SDS-PAGE on 12% gels and transferred to methanol-activated PVDF or 0.2 μm nitrocellulose membranes. Mouse anti-ToBRFV CP antibody (1:8,000) served as the primary antibody, followed by goat anti-mouse IgG secondary antibody (1:10,000; Transgen). Signals were visualized using the Invitrogen iBright™ 1500 imaging system.

**RNA preparation and quantitative real-time PCR analysis**

Total RNA was extracted using TRIzol reagent (Thermo Fisher Scientific) following the manufacturer’s protocol. Reverse transcription was performed with either TransScript® Uni All-in-One First-Strand cDNA Synthesis SuperMix (Transgen) or ReverTra Ace™ qPCR RT Master Mix (Toyobo). qRT-PCR was run on a QuantStudio™ 5 system using SYBR Green Master Mix (Vazyme), and analyzed by the 2^-ΔΔCT^ method. Primers targeted ToBRFV genomic RNA (ToBRFV-RdRp-qF/R), sub-genomic RNA (ToBRFV-CP-qF/R), and tomato actin (Slactin-F/R). Primer sequences are in Table S11.

**Cryo-SEM and TEM**

To examine morphological changes in trichomes and guard cells of JP and RG tomato plants after ToBRFV infection, Cryo-SEM was performed using a Hitachi SU8600 microscope. Leaf samples (1 cm × 1 cm) were excised, mounted, frozen at -140°C, sputter-coated with 6 nm gold-palladium, and imaged at 3 kV and -120°C. For pathological analysis, infected leaf tissues were fixed overnight at 4°C in 2.5% glutaraldehyde and 2% FAA, post-fixed in 1% OsO4 for 1 hour, dehydrated through graded ethanol and acetone series, and embedded in Spurr resin. Ultrathin sections were cut with a diamond knife, collected on copper grids or silicon wafers, and double-stained with uranyl acetate and lead citrate. Silicon wafer sections were observed by cryo-SEM, copper grid sections by Hitachi HT7800 TEM to detect virions. Imaging was done at 3 kV (cryo-SEM) and 80 kV (TEM) with random fields photographed at 100× and 1,000× (cryo-SEM) and 12,000× (TEM).

**Protoplast preparation for scRNA-seq**

Nine RG and nine JP tomato plants were inoculated with ToBRFV or pcb-GUS control. At 14 dpi, youngest systemic leaves were collected for protoplast preparation. Leaves were cut into 2–3 mm strips, placed in PBS, then transferred to an enzyme solution (1.25% cellulase R-10, 0.3% macerozyme R-10, 0.4 M mannitol, 10 mM CaCl2·2H_2_O, 20 mM KCl, 0.1% BSA, 20 mM MES, pH 5.7). Strips underwent 5-minute vacuum infiltration, then 3-hour incubation at 27°C with gentle shaking. Suspension was filtered through a 40 μm strainer and centrifuged at 100 g for 5 minutes at 4°C to collect protoplasts. After removing supernatant, protoplasts were washed twice with W5 solution (154 mM NaCl, 125 mM CaCl_2_·2H_2_O, 5 mM glucose, 2 mM MES, 5 mM KCl, pH 5.7). Protoplasts were resuspended in 0.8 M mannitol to reduce aggregation. Concentrations were determined microscopically, and viability assessed with 0.04% trypan blue staining.

**ScRNA library construction and sequencing**

Protoplast suspensions were loaded into the Chromium Controller (10× Genomics) to generate single-cell Gel Bead-In-Emulsions (GEMs). scRNA-seq libraries were prepared using Chromium Next GEM Single Cell 3’ Reagent Kits v3.1 per the vendor’s guide. Silane magnetic beads were used to remove residual reagents and primers. Full-length barcoded cDNAs were PCR-amplified for library construction. Libraries were adaptor-ligated, quality-checked with an Agilent 2100 Bioanalyzer, and sequenced on an Illumina NovaSeq 6000 platform (Gene Denovo) to generate paired-end 150 bp reads.

**Data quality control and gene expression quantification of scRNA**

10x Genomics Cell Ranger (v3.1.0) was used to convert BCL files to FASTQ, align reads, and quantify gene expression. Low-quality barcodes and UMIs were filtered before mapping to the reference genome. Reads with over 50% exon overlap were retained for UMI counting, with errors corrected. Valid cell barcodes were identified using the EmptyDrops method, and final output consisted of cell-by-gene expression matrices from UMI counts and barcode assignments.

**Cell clustering and cell type annotation and gene expression analysis in scRNA-seq**

For single-cell RNA-seq analysis, gene-by-cell matrices were imported into Seurat v3.1.1. Low-quality cells were filtered based on criteria such as UMIs ≥ 8,000, mitochondrial gene percentage ≥ 10%, and doublet GEMs. Data were normalized using the "LogNormalize" method, and Principal Component Analysis (PCA) was performed to reduce noise and identify significant principal components. Cells were clustered using Seurat’s graph-based method, with distances from selected PCs used to construct an shared-nearest neighbor (SNN) graph. Clusters were identified using the Louvain algorithm and visualized with distributed Stochastic Neighbor Embedding (t-SNE). Cell types were annotated using marker genes from Arabidopsis thaliana (https://www.tobaccodb.oRG/pcmdb/homePage) and homologous genes in tomato.

Expression values for each gene in a given cluster were compared to all other cells using the Wilcoxon rank-sum test. A hurdle model from the Model-based Analysis of Single-cell Transcriptomics (MAST) was then applied to identify differentially expressed genes within each cluster group. DEGs were identified based on a 1.28-fold overexpression in the target cluster, expression in >10% of cells, and a p-value < 0.05. GO enrichment analysis mapped genes to GO terms using the Gene Ontology database (http://www.geneontology.org/). Significantly enriched GO terms among DEGs were identified with the hypergeometric test. Pathway enrichment analysis of DEGs was performed using the KEGG database to identify biological pathways linked to differential gene expression in the clusters.

**Development trajectory and pseudotime analysis**

To identify BR-related genes, those associated with GO terms for positive/negative regulation of brassinosteroid biosynthesis (GO:0010422, GO:0010423) and signaling (GO:1900459, GO:1900458) were retrieved from the TAIR database and AmiGO 2.1 (https://amigo.geneontology.oRG/). Other hormone-related pathways were analyzed by identifying genes linked to the following GO terms: “induced systemic resistance, jasmonic acid-mediated signaling pathway” (GO:0009864), “regulation of jasmonic acid-mediated signaling pathway” (GO:2000022), “symbiont-mediated perturbation of host jasmonic acid signaling” (GO:0052088), “positive regulation of salicylic acid-mediated signaling pathway” (GO:0080151), “negative regulation of systemic acquired resistance” (GO:0010113), “ethylene-activated signaling pathway” (GO:0009873), “negative regulation of ethylene-activated signaling pathway” (GO:0010105), “positive regulation of abscisic acid-activated signaling pathway” (GO:0009788), “negative regulation of abscisic acid-activated signaling pathway” (GO:0009788), “positive regulation of cytokinin-mediated signaling pathway” (GO:0001961), “negative regulation of cytokinin-mediated signaling pathway” (GO:0001960), “positive regulation of auxin-mediated signaling pathway” (GO:0010929), “negative regulation of auxin-mediated signaling pathway” (GO:0010930), “positive regulation of gibberellic acid-mediated signaling pathway” (GO:0009939), and “negative regulation of gibberellic acid-mediated signaling pathway” (GO:0009938).

Gene annotations were retrieved from the TAIR database and AmiGO 2.1. The combined gene lists were analyzed using the “AddModuleScore” function to calculate relative expression of BR-related genes in each cell, quantifying pathway activity for BR, JA, SA, ethylene, ABA, cytokinin, auxin, and gibberellin at the single-cell level. Enrichment analyses were performed on the Omicsmart platform with a false discovery rate (FDR) threshold < 0.05 to identify significantly enriched GO terms.

**EBL treatment**

A 3 mg/mL EBL stock solution was prepared by dissolving EBL in ethanol and adjusting the volume to 1 mL with distilled water. This stock was diluted to 0.3 mg/L with 0.1% (v/v) Tween-80 to create the working solution. Approximately 5 mL of the solution was sprayed evenly on the leaves of each plant, ensuring full coverage. Tomato seedlings (JP or RG) inoculated with ToBRFV and verified for gene silencing were divided into two groups: one sprayed with 50 mL of distilled water with 0.01% Tween-80 (buffer control), and the other with 50 mL of 0.3 mg/L EBL solution. Treatment started 24 hours after ToBRFV inoculation and was applied once daily for 12 days.

**Measurement of endogenous BRs**

HPLC-grade BR was purchased from Shanghai Yuanye Bio-Technology Co Ltd (Shanghai, China), while HPLC-grade methanol and ethyl acetate were from Aladdin (Shanghai, China). For the extraction and purification of plant hormones, approximately 0.1 g of fresh tomato leaves were frozen in liquid nitrogen and ground into powder. A 1 mL aliquot of pre-cooled ethyl acetate was added, shaken for 15 minutes, and placed on a swing bed overnight at 4°C. The mixture was then centrifuged at 12,000 rpm for 10 minutes, and the supernatant was transferred to 2 mL tubes and evaporated at room temperature for 120 minutes. Each sample was re-solubilized in 50% methanol (100 μL), filtered using a 0.45 μm microfilter, and prepared for HPLC-MS/MS analysis. The mixtures of compounds were separated and analyzed by using an ExionLC™ AC (AB Sciex) and SCIEX Triple Quad™ 4500 (AB Sciex) equipped with a Kinetex polar C18 column (30 mm × 2.1 mm, 2.6 μm particle size, Xiamen, China). The mobile phase consisted of acetonitrile with 0.1% formic acid in ultrapure water (Phase A) and acetonitrile (Phase B), pumped at a flow rate of 0.3 mL/min. The gradient elution program was as follows: 0.0-0.5 min, 100% A; 0.5-4.0 min, 100%-10% A; 4.0-6.0 min, 10% A; 6.0-6.1 min, 10%-100% A; 6.1-7.5 min, 100% A for column equilibration. The column oven temperature was maintained at 40°C, and the autosampler temperature was set to 15°C. The injection volume was 10 μL. Mass spectrometry analysis was conducted using an AB SCIEX Triple Quad™ 4500 with an electrospray ionization source (ESI) in positive mode (ESI+). Multiple reaction monitoring (MRM) was used for BRs, with typical MS/MS instrument parameters shown in Figure S7A. Instrument control, data acquisition, and analysis were managed using Analyst 1.6.3 software (AB Sciex Corp., USA) and SCIEX OS-Q (AB Sciex Corp., USA).

**Whole-genome resequencing**

Young leaves from healthy RG and JP tomato plants were collected, immediately frozen in liquid nitrogen, and stored at −80 °C until use. Total genomic DNA was extracted using the EZgene™ CP Plant Miniprep Kit (Biomiga, USA) following the manufacturer’s instructions. For each sample, 0.2 μg of DNA was used as input material for library preparation. Genomic DNA was fragmented by sonication to an average size of 350 bp, followed by end polishing, A-tailing, and ligation with full-length Illumina adapters. The ligated fragments were size-selected and amplified by PCR. PCR products were purified using the AMPure XP system (Beverly, USA). Library quality was assessed using the Agilent 5400 system (Agilent, USA) and quantified by qPCR (1.5 nM). Qualified libraries were pooled and sequenced on an Illumina platform with the PE150 strategy. The main steps of data analysis were as follows: (1) quality control of the raw sequencing data to obtain clean reads, (2) alignment of clean reads to the reference genome, and (3) detection and functional annotation of SNPs, InDels, and CNVs based on the alignment results.

**References**

Han, K., Jia, Z., Zhang, Y., Zhou, H., Bu, S., Chen, J., Yan, D., Qi, R., Yan, F., and Wu, J. (2023). Chloroplast clustering around the nucleus induced by OMP24 overexpression unexpectedly promoted PSTVd infection in Nicotiana benthamiana. *Mol. Plant Pathol.* **24**, 1552-1559.
