# Supplementary material for: Dissecting the Cell‐Type‐Specific Response to an Emerging Tobamovirus in Tomato Reveals Cultivar‐Dependent Involvement of Brassinosteroid Signalling
Source: Plant Biotechnol J. 2026 Jan 21;24(5):3106–24. doi: 10.1111/pbi.70559 (PMC13110166; doi:10.1111/pbi.70559)
Supplement: Supplementary file 2 — Figure S1: Quality control of single‐cell sequencing data and expression level quantification. Figure S2: Expression patterns of potential novel marker genes in tomato leaf cells. Figure S3: DEGs and KEGG pathway enrichment in mock‐ and ToBRFV‐infected samples. Figure S4: Differentiation trajectory of mesophyll cells. Figure S5: Hormone signalling dynamics in tomato mesophyll cells during JP ‐mediated defence against ToBRFV infection. Figure S6: Phenotypic characterisation of BR‐related gene‐silenced tomato plants. Figure S7: The changes of endogenous BRs levels in tomato leaves upon ToBRFV infection in two tomato cultivars. Figure S8: Phenotypic characterisation of BR‐related gene‐silenced tomato plants treated with EBL. Figure S9: Identification and functional annotation of SNPs and InDels in two tomato cultivars. Figure S10: Characterisation of genome‐wide genetic variations in two tomato cultivars. [file PBI-24-3106-s006.pptx]

## Slide 1
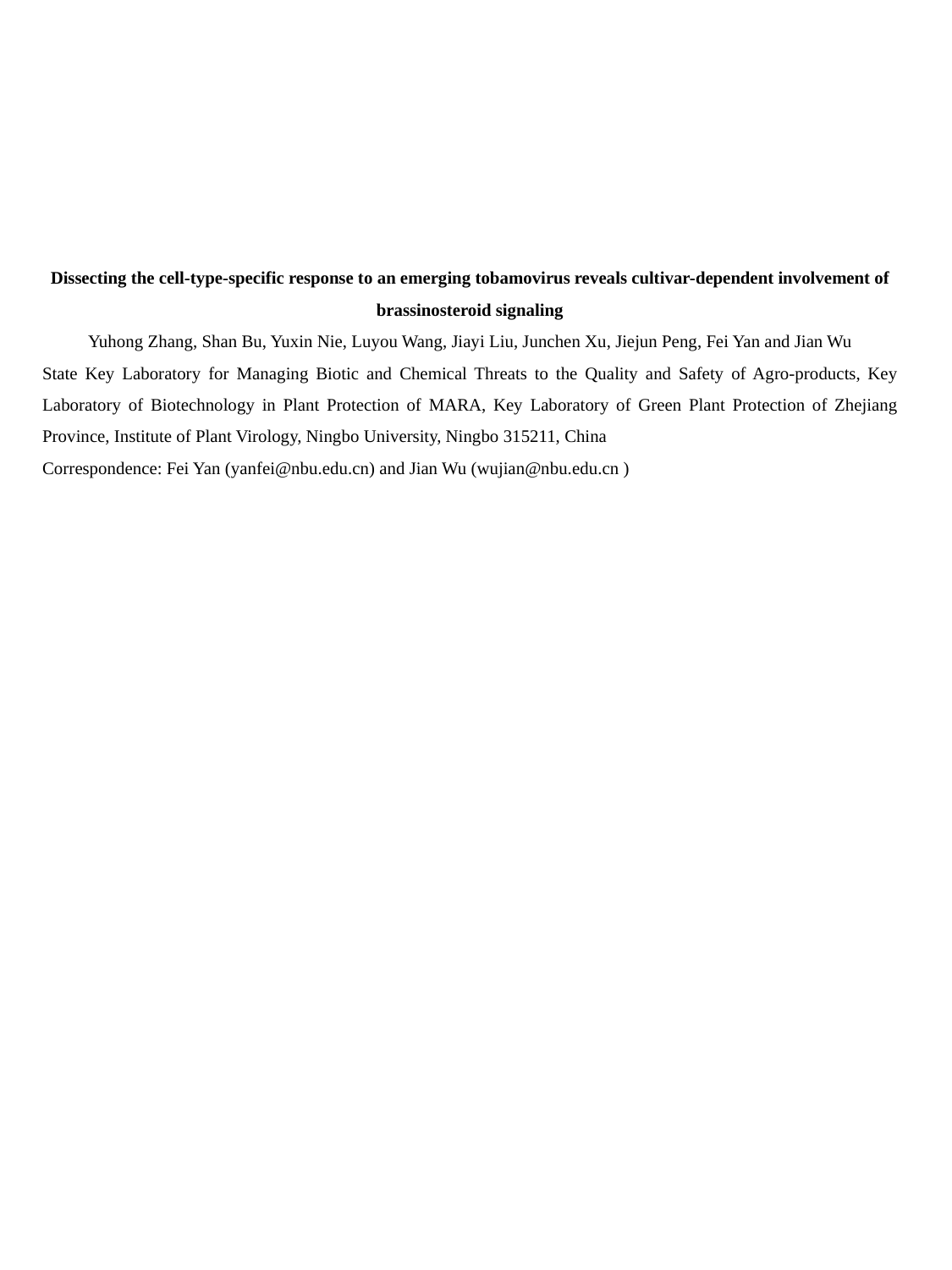

Dissecting the cell-type-specific response to an emerging tobamovirus reveals cultivar-dependent involvement of brassinosteroid signaling
Yuhong Zhang, Shan Bu, Yuxin Nie, Luyou Wang, Jiayi Liu, Junchen Xu, Jiejun Peng, Fei Yan and Jian Wu
State Key Laboratory for Managing Biotic and Chemical Threats to the Quality and Safety of Agro-products, Key Laboratory of Biotechnology in Plant Protection of MARA, Key Laboratory of Green Plant Protection of Zhejiang Province, Institute of Plant Virology, Ningbo University, Ningbo 315211, China
Correspondence: Fei Yan (yanfei@nbu.edu.cn) and Jian Wu (wujian@nbu.edu.cn )

## Slide 2
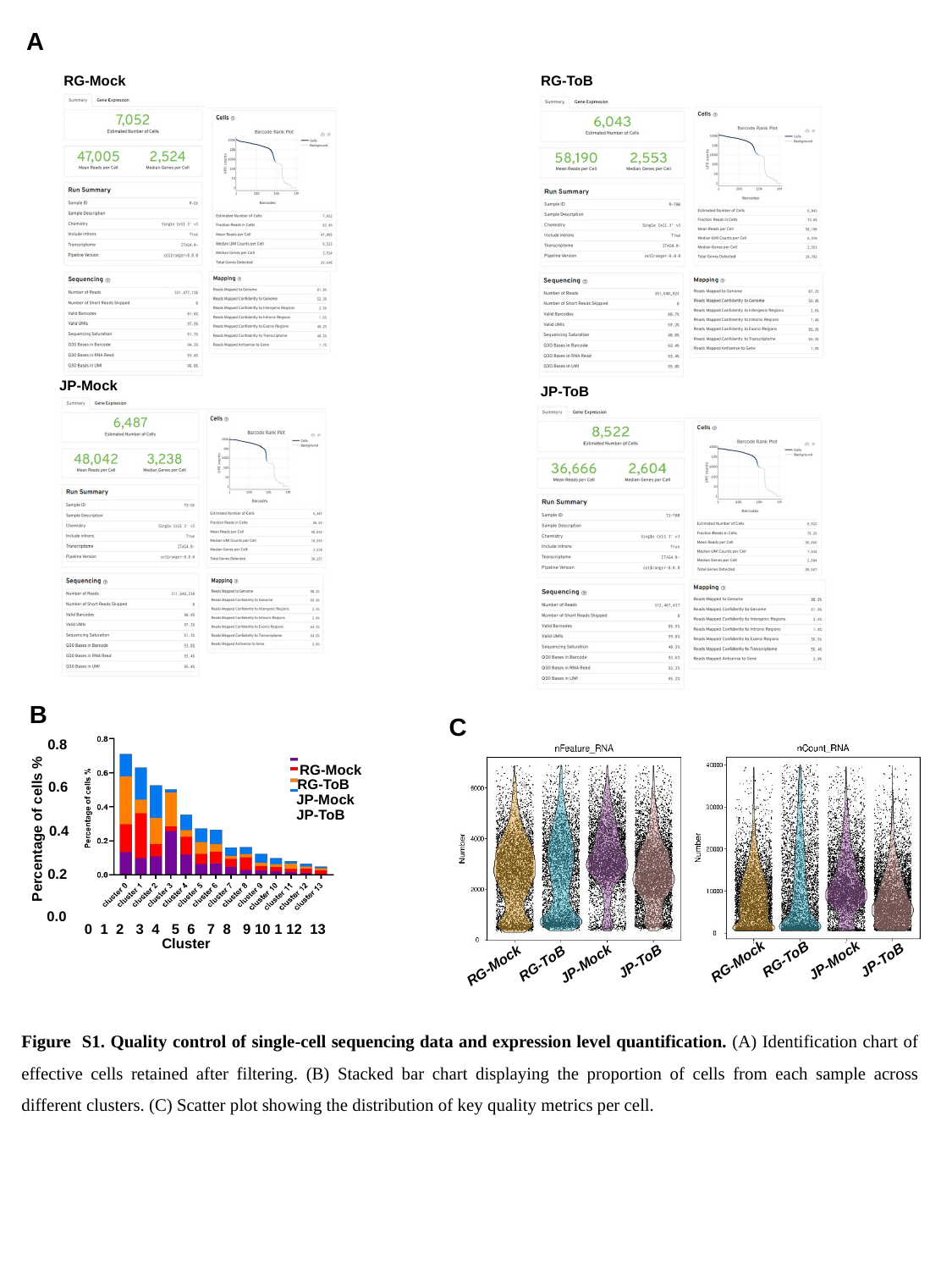

A
RG-Mock
RG-ToB
JP-Mock
JP-ToB
B
C
0.8
JP-Mock
JP-ToB
RG-Mock
RG-ToB
JP-ToB
JP-Mock
RG-Mock
RG-ToB
RG-Mock
RG-ToB
0.6
JP-Mock
JP-ToB
Percentage of cells %
0.4
0.2
0.0
0 1 2 3 4 5 6 7 8 9 10 1 12 13
Cluster
Figure S1. Quality control of single-cell sequencing data and expression level quantification. (A) Identification chart of effective cells retained after filtering. (B) Stacked bar chart displaying the proportion of cells from each sample across different clusters. (C) Scatter plot showing the distribution of key quality metrics per cell.

## Slide 3
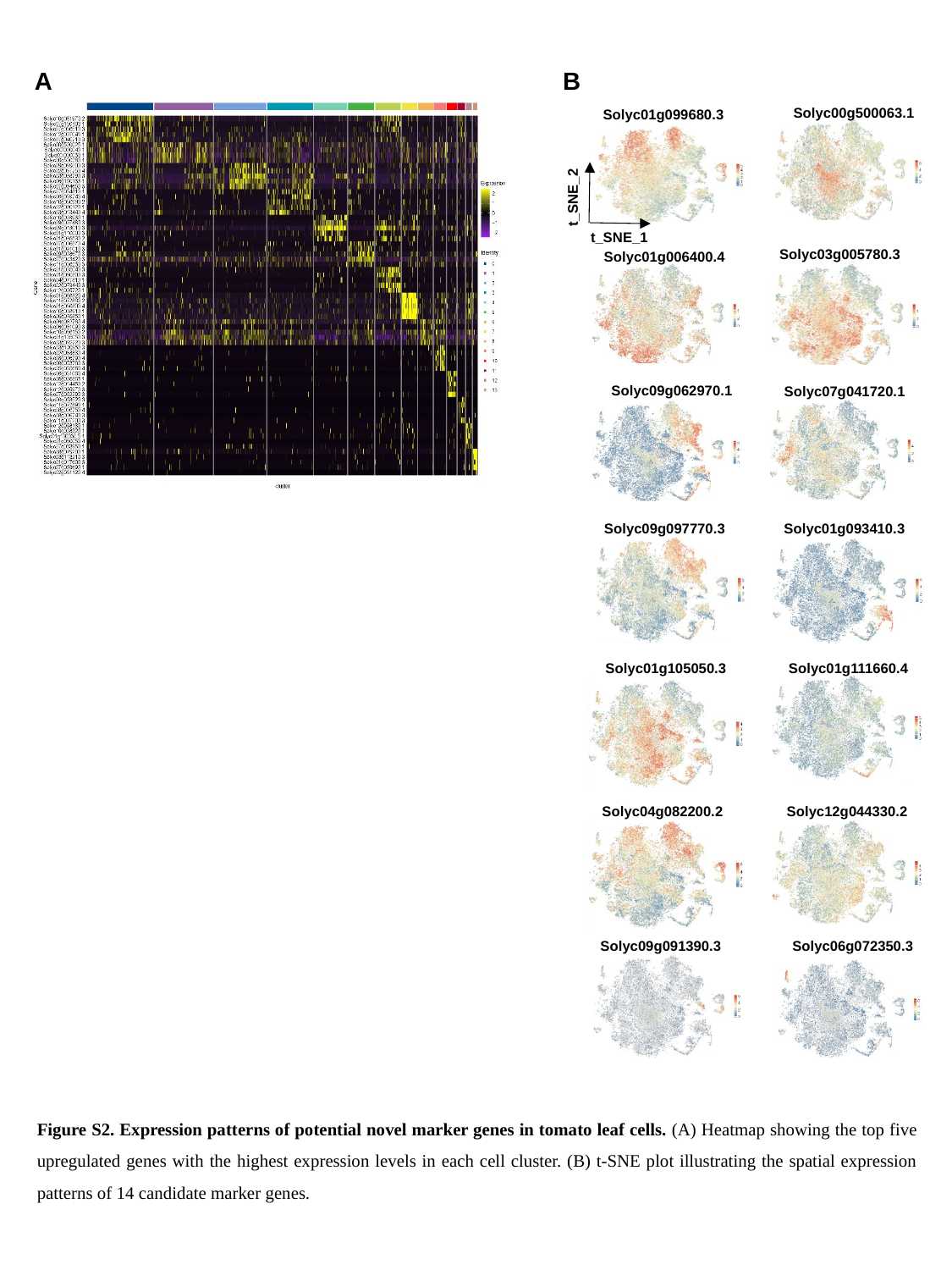

A
B
Solyc00g500063.1
Solyc01g099680.3
t_SNE_2
t_SNE_1
Solyc03g005780.3
Solyc01g006400.4
Solyc09g062970.1
Solyc07g041720.1
Solyc01g093410.3
Solyc09g097770.3
Solyc01g111660.4
Solyc01g105050.3
Solyc04g082200.2
Solyc12g044330.2
Solyc09g091390.3
Solyc06g072350.3
Figure S2. Expression patterns of potential novel marker genes in tomato leaf cells. (A) Heatmap showing the top five upregulated genes with the highest expression levels in each cell cluster. (B) t-SNE plot illustrating the spatial expression patterns of 14 candidate marker genes.

## Slide 4
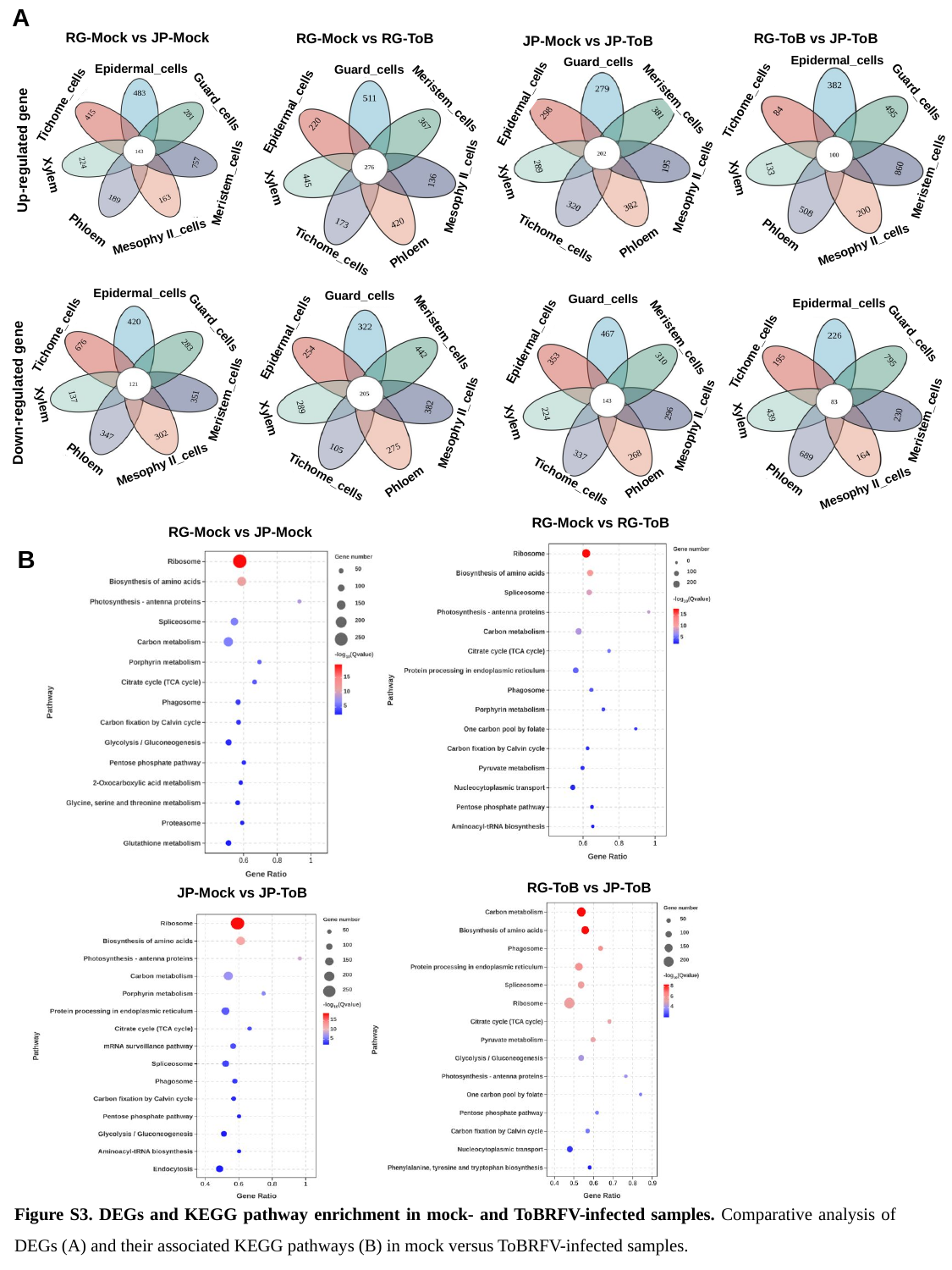

A
RG-Mock vs JP-Mock
RG-ToB vs JP-ToB
RG-Mock vs RG-ToB
JP-Mock vs JP-ToB
Epidermal_cells
Tichome_cells
Guard_cells
Meristem_cells
Xylem
Mesophy II_cells
Phloem
Guard_cells
Epidermal_cells
Meristem_cells
Mesophy II_cells
Xylem
Phloem
Tichome_cells
Epidermal_cells
Tichome_cells
Guard_cells
Meristem_cells
Xylem
Phloem
Mesophy II_cells
Guard_cells
Meristem_cells
Epidermal_cells
Mesophy II_cells
Xylem
Phloem
Tichome_cells
Up-regulated gene
Guard_cells
Epidermal_cells
Meristem_cells
Mesophy II_cells
Xylem
Phloem
Tichome_cells
Guard_cells
Epidermal_cells
Meristem_cells
Mesophy II_cells
Xylem
Phloem
Tichome_cells
Epidermal_cells
Guard_cells
Tichome_cells
Meristem_cells
Xylem
Phloem
Mesophy II_cells
Epidermal_cells
Guard_cells
Tichome_cells
Meristem_cells
Xylem
Phloem
Mesophy II_cells
Down-regulated gene
RG-Mock vs RG-ToB
RG-Mock vs JP-Mock
B
RG-ToB vs JP-ToB
JP-Mock vs JP-ToB
Figure S3. DEGs and KEGG pathway enrichment in mock- and ToBRFV-infected samples. Comparative analysis of DEGs (A) and their associated KEGG pathways (B) in mock versus ToBRFV-infected samples.

## Slide 5
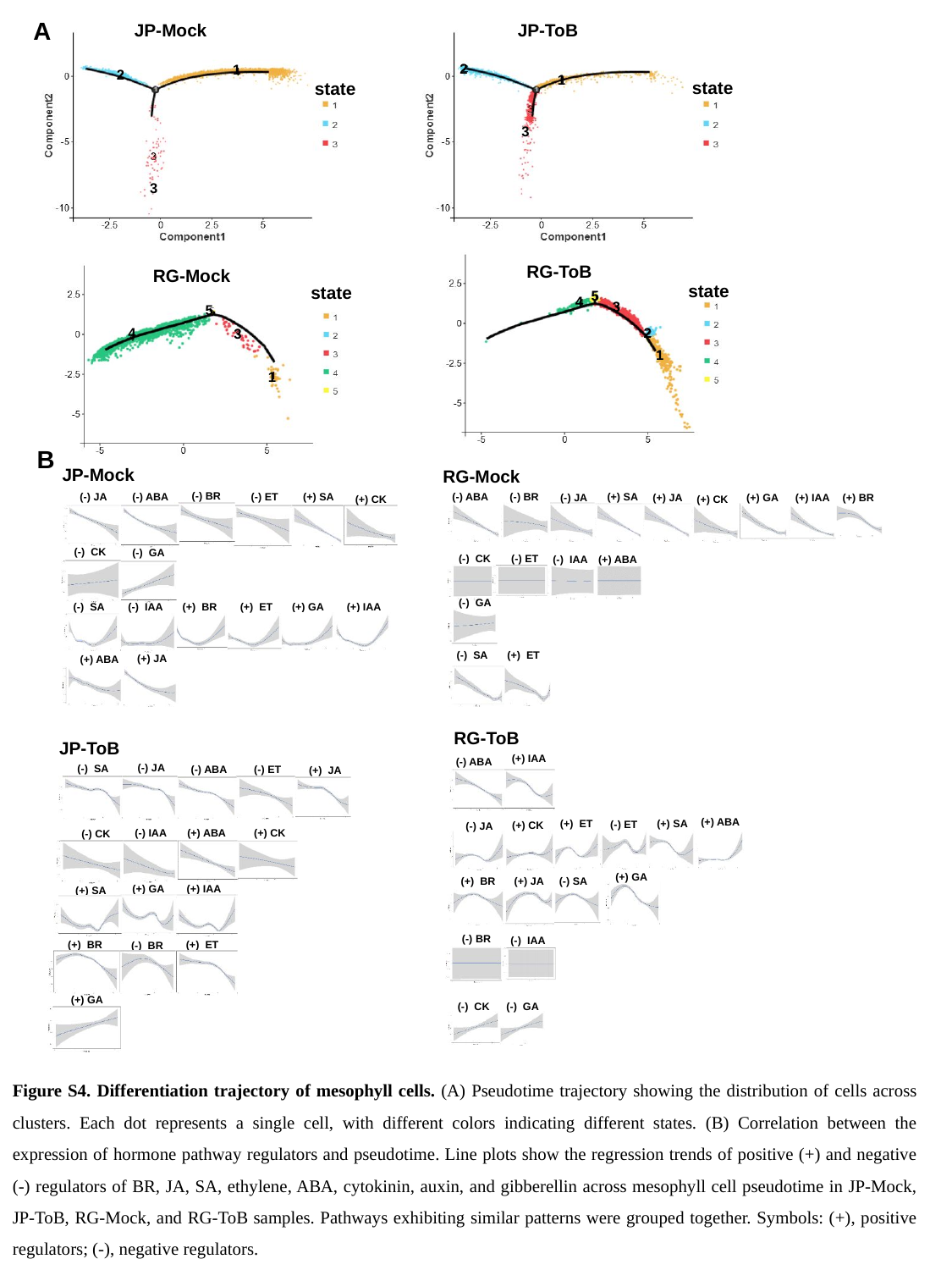

A
JP-Mock
JP-ToB
2
1
2
1
state
state
3
3
RG-ToB
RG-Mock
state
state
5
4
3
5
2
4
3
1
1
B
JP-Mock
RG-Mock
(-) ABA
(-) BR
(+) SA
(+) BR
(-) JA
(+) JA
(+) GA
(+) IAA
(+) CK
(-) CK
(-) ET
(-) IAA
(+) ABA
(-) GA
(+) ET
(-) SA
(-) BR
(+) SA
(-) ABA
(-) ET
(-) JA
(+) CK
(-) CK
(-) GA
(+) IAA
(+) ET
(+) BR
(-) IAA
(-) SA
(+) GA
(+) JA
(+) ABA
RG-ToB
(+) IAA
(-) ABA
(+) ABA
(+) SA
(+) ET
(-) ET
(+) CK
(-) JA
(+) BR
(+) JA
(-) SA
(-) BR
(-) IAA
(-) CK
(-) GA
JP-ToB
(-) JA
(-) SA
(-) ABA
(-) ET
(+) JA
(+) ABA
(+) CK
(-) IAA
(-) CK
(+) GA
(+) IAA
(+) SA
(+) BR
(+) ET
(-) BR
(+) GA
(+) GA
Figure S4. Differentiation trajectory of mesophyll cells. (A) Pseudotime trajectory showing the distribution of cells across clusters. Each dot represents a single cell, with different colors indicating different states. (B) Correlation between the expression of hormone pathway regulators and pseudotime. Line plots show the regression trends of positive (+) and negative (-) regulators of BR, JA, SA, ethylene, ABA, cytokinin, auxin, and gibberellin across mesophyll cell pseudotime in JP-Mock, JP-ToB, RG-Mock, and RG-ToB samples. Pathways exhibiting similar patterns were grouped together. Symbols: (+), positive regulators; (-), negative regulators.

## Slide 6
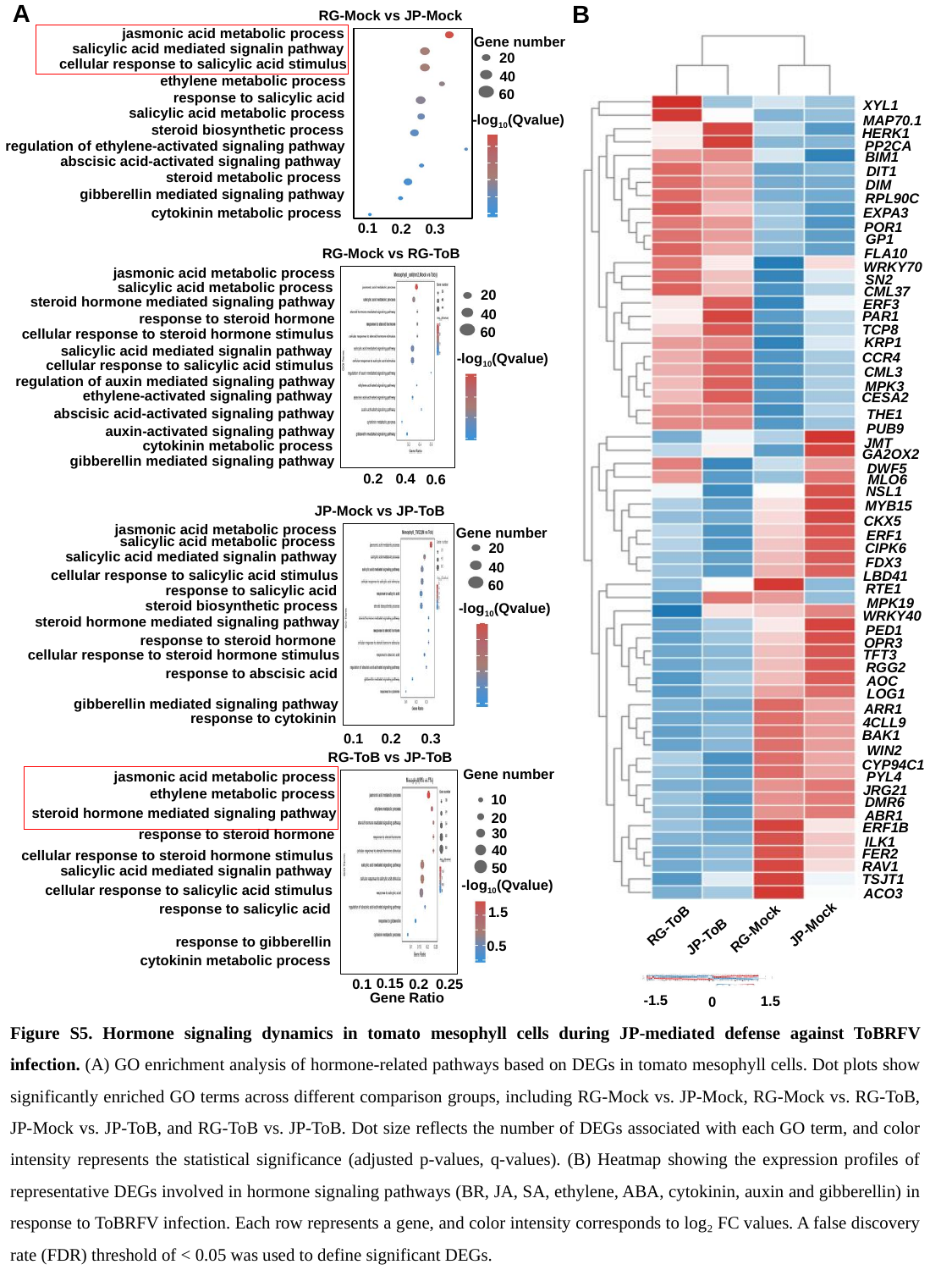

A
B
RG-Mock vs JP-Mock
jasmonic acid metabolic process
0.1
0.2
0.3
salicylic acid mediated signalin pathway
cellular response to salicylic acid stimulus
ethylene metabolic process
response to salicylic acid
salicylic acid metabolic process
steroid biosynthetic process
abscisic acid-activated signaling pathway
steroid metabolic process
gibberellin mediated signaling pathway
cytokinin metabolic process
Gene number
20
40
60
-log10(Qvalue)
4
3
2
1
0
regulation of ethylene-activated signaling pathway
XYL1
MAP70.1
HERK1
PP2CA
BIM1
DIT1
DIM
RPL90C
EXPA3
POR1
GP1
FLA10
WRKY70
SN2
CML37
ERF3
PAR1
TCP8
KRP1
CCR4
CML3
MPK3
CESA2
THE1
PUB9
JMT
GA2OX2
DWF5
MLO6
NSL1
MYB15
CKX5
ERF1
CIPK6
FDX3
LBD41
RTE1
MPK19
WRKY40
PED1
OPR3
TFT3
RGG2
AOC
LOG1
ARR1
4CLL9
BAK1
WIN2
CYP94C1
PYL4
JRG21
DMR6
ABR1
ERF1B
ILK1
FER2
RAV1
TSJT1
ACO3
JP-Mock
JP-ToB
RG-Mock
RG-ToB
1.5
0
-1.5
RG-Mock vs RG-ToB
jasmonic acid metabolic process
salicylic acid metabolic process
response to steroid hormone
cellular response to steroid hormone stimulus
salicylic acid mediated signalin pathway
regulation of auxin mediated signaling pathway
auxin-activated signaling pathway
cytokinin metabolic process
gibberellin mediated signaling pathway
20
steroid hormone mediated signaling pathway
40
60
-log10(Qvalue)
cellular response to salicylic acid stimulus
3
2
1
0
ethylene-activated signaling pathway
abscisic acid-activated signaling pathway
0.2
0.4
0.6
JP-Mock vs JP-ToB
jasmonic acid metabolic process
Gene number
20
40
60
-log10(Qvalue)
4
3
2
1
0
5
salicylic acid metabolic process
salicylic acid mediated signalin pathway
cellular response to salicylic acid stimulus
response to salicylic acid
steroid biosynthetic process
steroid hormone mediated signaling pathway
response to steroid hormone
cellular response to steroid hormone stimulus
response to abscisic acid
gibberellin mediated signaling pathway
response to cytokinin
0.2
0.1
0.3
RG-ToB vs JP-ToB
Gene number
jasmonic acid metabolic process
ethylene metabolic process
10
steroid hormone mediated signaling pathway
20
30
response to steroid hormone
40
cellular response to steroid hormone stimulus
50
salicylic acid mediated signalin pathway
-log10(Qvalue)
cellular response to salicylic acid stimulus
response to salicylic acid
1.5
1
0.5
0
response to gibberellin
cytokinin metabolic process
Gene Ratio
0.15
0.1
0.2
0.25
Figure S5. Hormone signaling dynamics in tomato mesophyll cells during JP-mediated defense against ToBRFV infection. (A) GO enrichment analysis of hormone-related pathways based on DEGs in tomato mesophyll cells. Dot plots show significantly enriched GO terms across different comparison groups, including RG-Mock vs. JP-Mock, RG-Mock vs. RG-ToB, JP-Mock vs. JP-ToB, and RG-ToB vs. JP-ToB. Dot size reflects the number of DEGs associated with each GO term, and color intensity represents the statistical significance (adjusted p-values, q-values). (B) Heatmap showing the expression profiles of representative DEGs involved in hormone signaling pathways (BR, JA, SA, ethylene, ABA, cytokinin, auxin and gibberellin) in response to ToBRFV infection. Each row represents a gene, and color intensity corresponds to log₂ FC values. A false discovery rate (FDR) threshold of < 0.05 was used to define significant DEGs.

## Slide 7
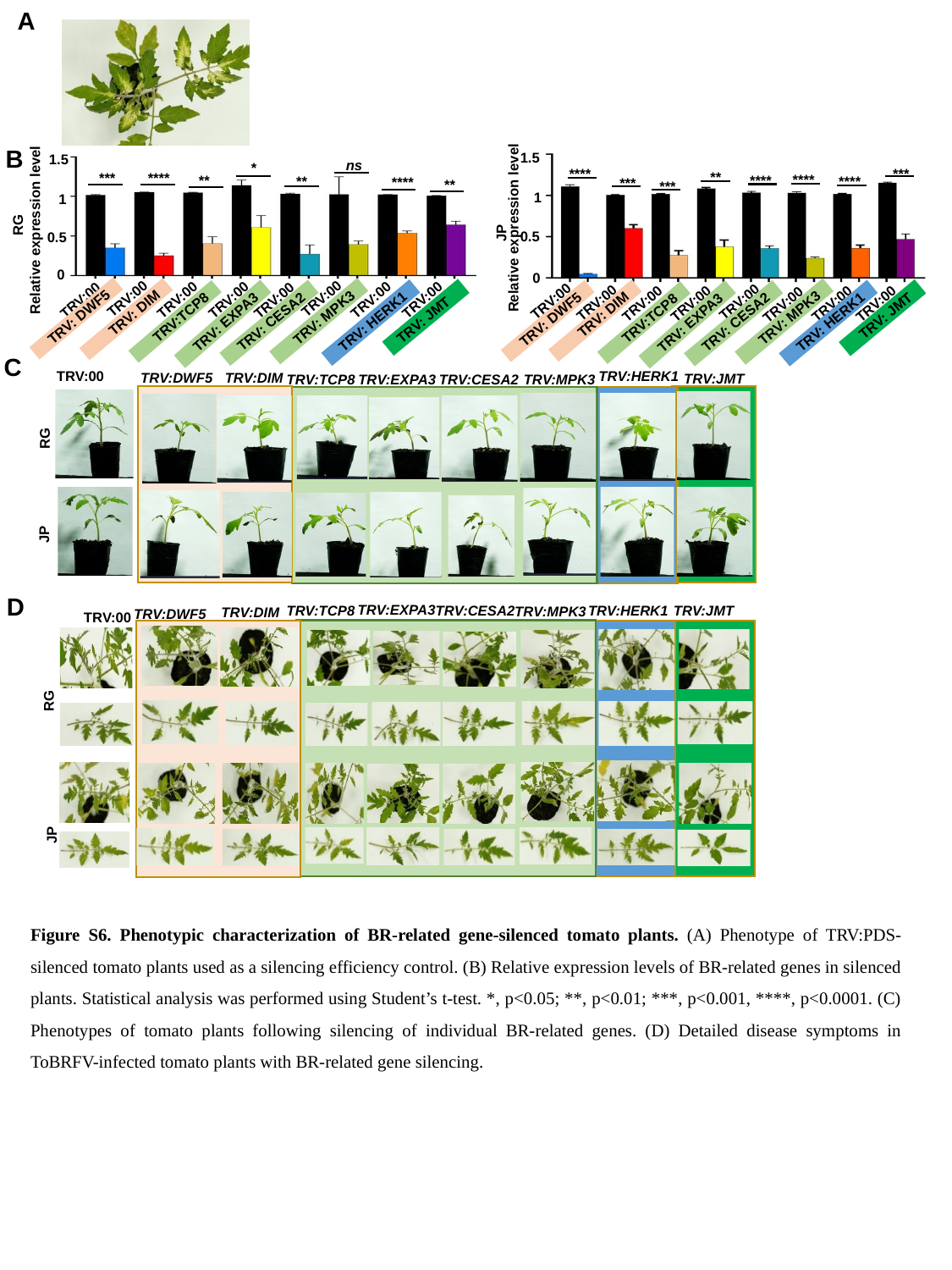

A
1.5
1
0.5
0
***
****
**
****
****
****
***
***
JP
Relative expression level
TRV:00
TRV:00
TRV:00
TRV:00
TRV:00
TRV:00
TRV:00
TRV:00
TRV: JMT
TRV: DIM
TRV: MPK3
TRV: DWF5
TRV: HERK1
TRV: CESA2
TRV:TCP8
TRV: EXPA3
1.5
1
0.5
0
ns
*
***
****
**
**
****
**
RG
Relative expression level
TRV:00
TRV:00
TRV:00
TRV:00
TRV:00
TRV:00
TRV:00
TRV:00
TRV: JMT
TRV: DWF5
TRV: DIM
TRV: MPK3
TRV:TCP8
TRV: HERK1
TRV: CESA2
TRV: EXPA3
B
C
TRV:HERK1
TRV:00
TRV:DWF5
TRV:DIM
TRV:JMT
TRV:TCP8
TRV:MPK3
TRV:EXPA3
TRV:CESA2
RG
JP
D
TRV:EXPA3
TRV:HERK1
TRV:CESA2
TRV:TCP8
TRV:JMT
TRV:MPK3
TRV:DIM
TRV:DWF5
TRV:00
RG
JP
Figure S6. Phenotypic characterization of BR-related gene-silenced tomato plants. (A) Phenotype of TRV:PDS-silenced tomato plants used as a silencing efficiency control. (B) Relative expression levels of BR-related genes in silenced plants. Statistical analysis was performed using Student’s t-test. *, p<0.05; **, p<0.01; ***, p<0.001, ****, p<0.0001. (C) Phenotypes of tomato plants following silencing of individual BR-related genes. (D) Detailed disease symptoms in ToBRFV-infected tomato plants with BR-related gene silencing.

## Slide 8
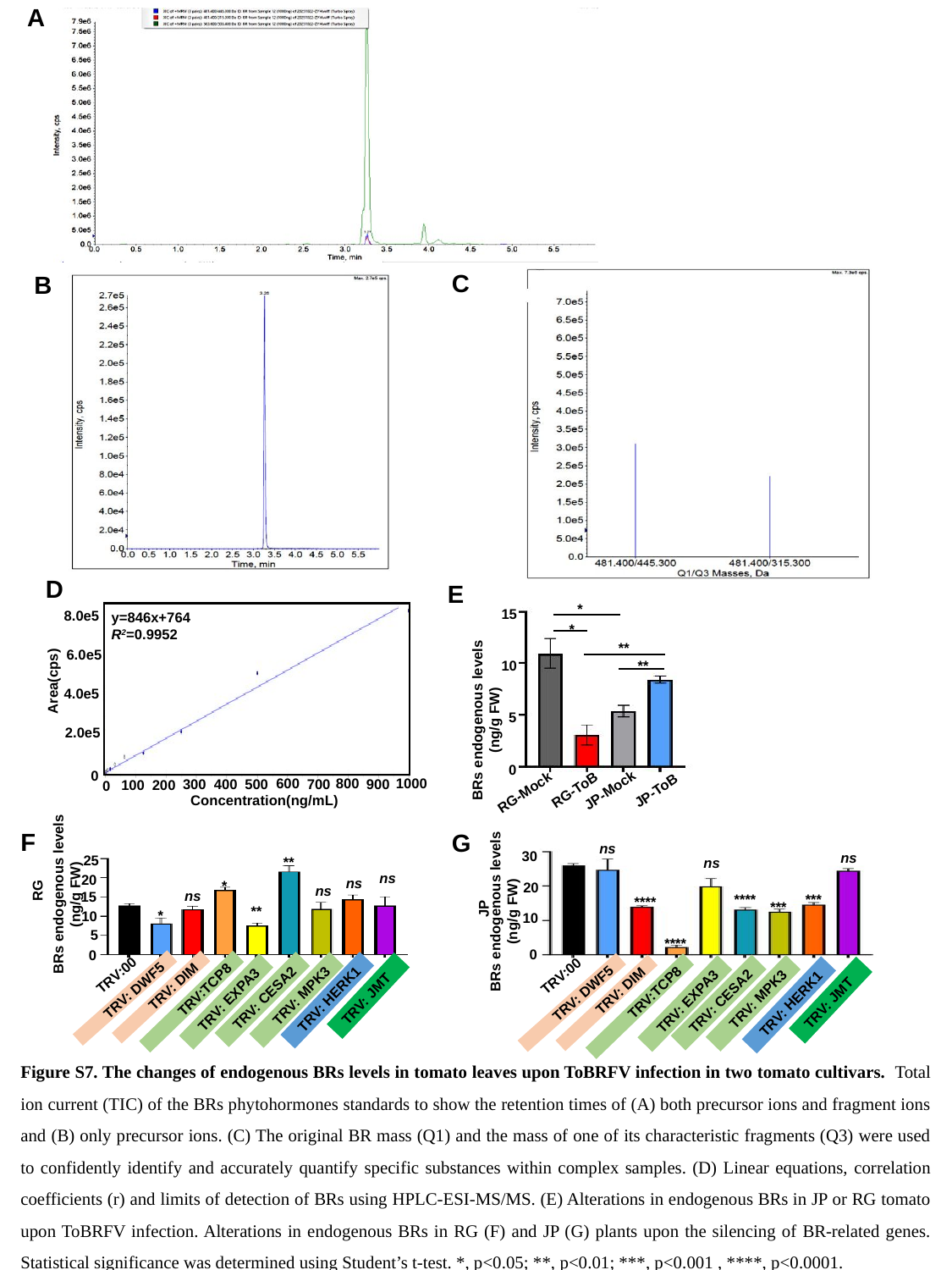

A
C
B
D
E
*
15
*
10
5
0
JP-Mock
JP-ToB
RG-ToB
RG-Mock
8.0e5
y=846x+764
R2=0.9952
6.0e5
Area(cps)
4.0e5
2.0e5
0
1000
600
800
900
300
500
700
400
200
100
0
Concentration(ng/mL)
**
**
BRs endogenous levels (ng/g FW)
F
G
ns
30
ns
ns
20
****
****
***
10
****
0
25
**
ns
20
ns
*
ns
ns
15
**
*
10
5
0
TRV:00
TRV: JMT
TRV: DIM
TRV: DWF5
TRV: CESA2
TRV: MPK3
TRV: HERK1
TRV: EXPA3
TRV:TCP8
RG
BRs endogenous levels (ng/g FW)
JP
***
BRs endogenous levels (ng/g FW)
TRV:00
TRV: JMT
TRV: CESA2
TRV: MPK3
TRV: DWF5
TRV: EXPA3
TRV: DIM
TRV: HERK1
TRV:TCP8
Figure S7. The changes of endogenous BRs levels in tomato leaves upon ToBRFV infection in two tomato cultivars. Total ion current (TIC) of the BRs phytohormones standards to show the retention times of (A) both precursor ions and fragment ions and (B) only precursor ions. (C) The original BR mass (Q1) and the mass of one of its characteristic fragments (Q3) were used to confidently identify and accurately quantify specific substances within complex samples. (D) Linear equations, correlation coefficients (r) and limits of detection of BRs using HPLC‐ESI‐MS/MS. (E) Alterations in endogenous BRs in JP or RG tomato upon ToBRFV infection. Alterations in endogenous BRs in RG (F) and JP (G) plants upon the silencing of BR-related genes. Statistical significance was determined using Student’s t-test. *, p<0.05; **, p<0.01; ***, p<0.001 , ****, p<0.0001.

## Slide 9
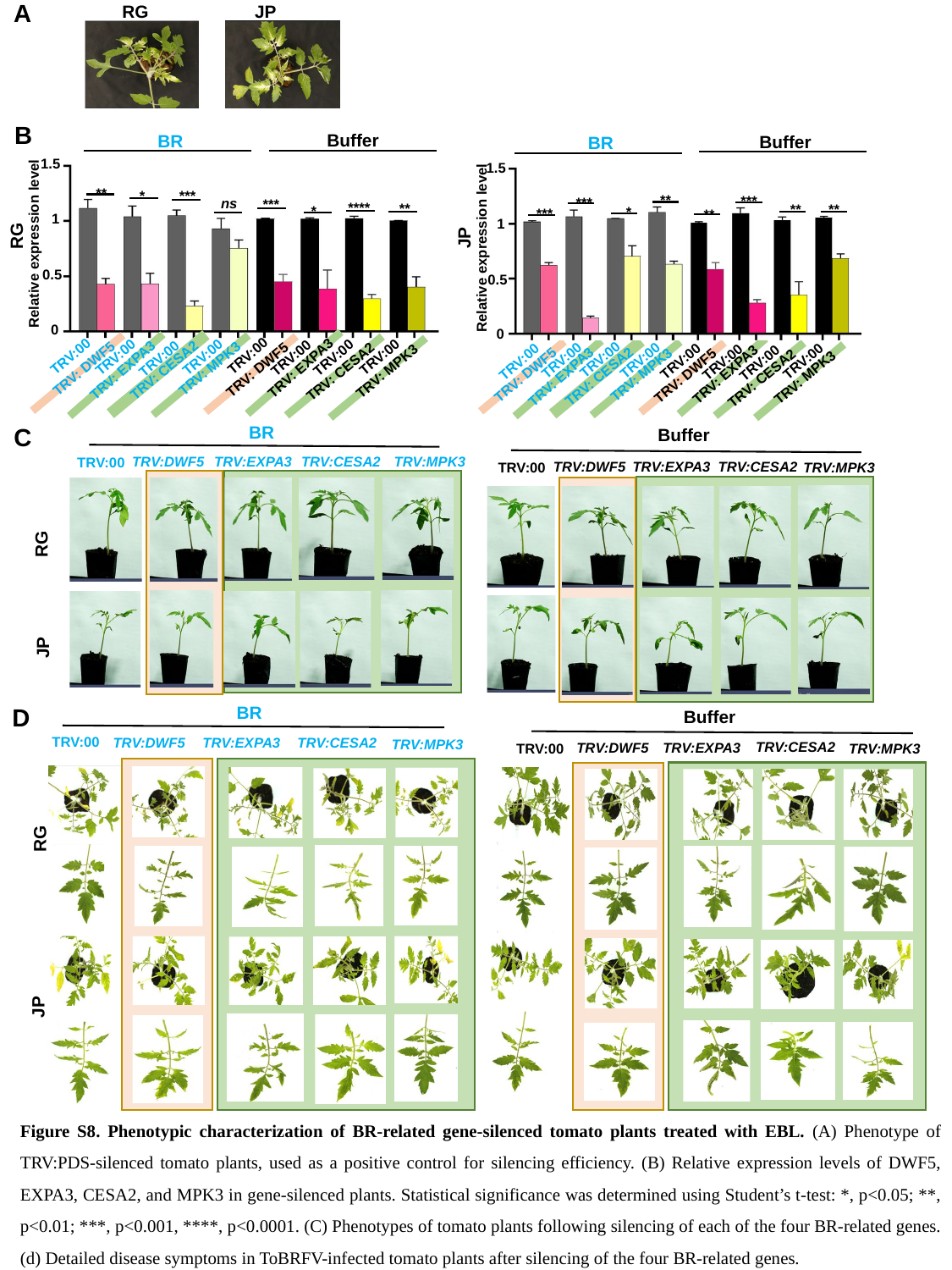

A
RG
JP
B
Buffer
BR
1.5
1
0.5
0
**
*
***
ns
***
****
**
*
RG
Relative expression level
TRV:00
TRV:00
TRV:00
TRV:00
TRV:00
TRV:00
TRV:00
TRV:00
TRV: CESA2
TRV: EXPA3
TRV: DWF5
TRV: DWF5
TRV: EXPA3
TRV: MPK3
TRV: CESA2
TRV: MPK3
Buffer
BR
1.5
1
0.5
0
**
***
***
**
**
*
***
**
JP
Relative expression level
TRV:00
TRV:00
TRV:00
TRV:00
TRV:00
TRV:00
TRV:00
TRV:00
TRV: CESA2
TRV: EXPA3
TRV: DWF5
TRV: DWF5
TRV: EXPA3
TRV: MPK3
TRV: CESA2
TRV: MPK3
BR
TRV:MPK3
TRV:DWF5
TRV:EXPA3
TRV:CESA2
TRV:00
C
Buffer
TRV:CESA2
TRV:DWF5
TRV:EXPA3
TRV:00
TRV:MPK3
RG
JP
BR
TRV:00
TRV:DWF5
TRV:EXPA3
TRV:CESA2
TRV:MPK3
D
Buffer
TRV:CESA2
TRV:DWF5
TRV:EXPA3
TRV:00
TRV:MPK3
RG
JP
Figure S8. Phenotypic characterization of BR-related gene-silenced tomato plants treated with EBL. (A) Phenotype of TRV:PDS-silenced tomato plants, used as a positive control for silencing efficiency. (B) Relative expression levels of DWF5, EXPA3, CESA2, and MPK3 in gene-silenced plants. Statistical significance was determined using Student’s t-test: *, p<0.05; **, p<0.01; ***, p<0.001, ****, p<0.0001. (C) Phenotypes of tomato plants following silencing of each of the four BR-related genes. (d) Detailed disease symptoms in ToBRFV-infected tomato plants after silencing of the four BR-related genes.

## Slide 10
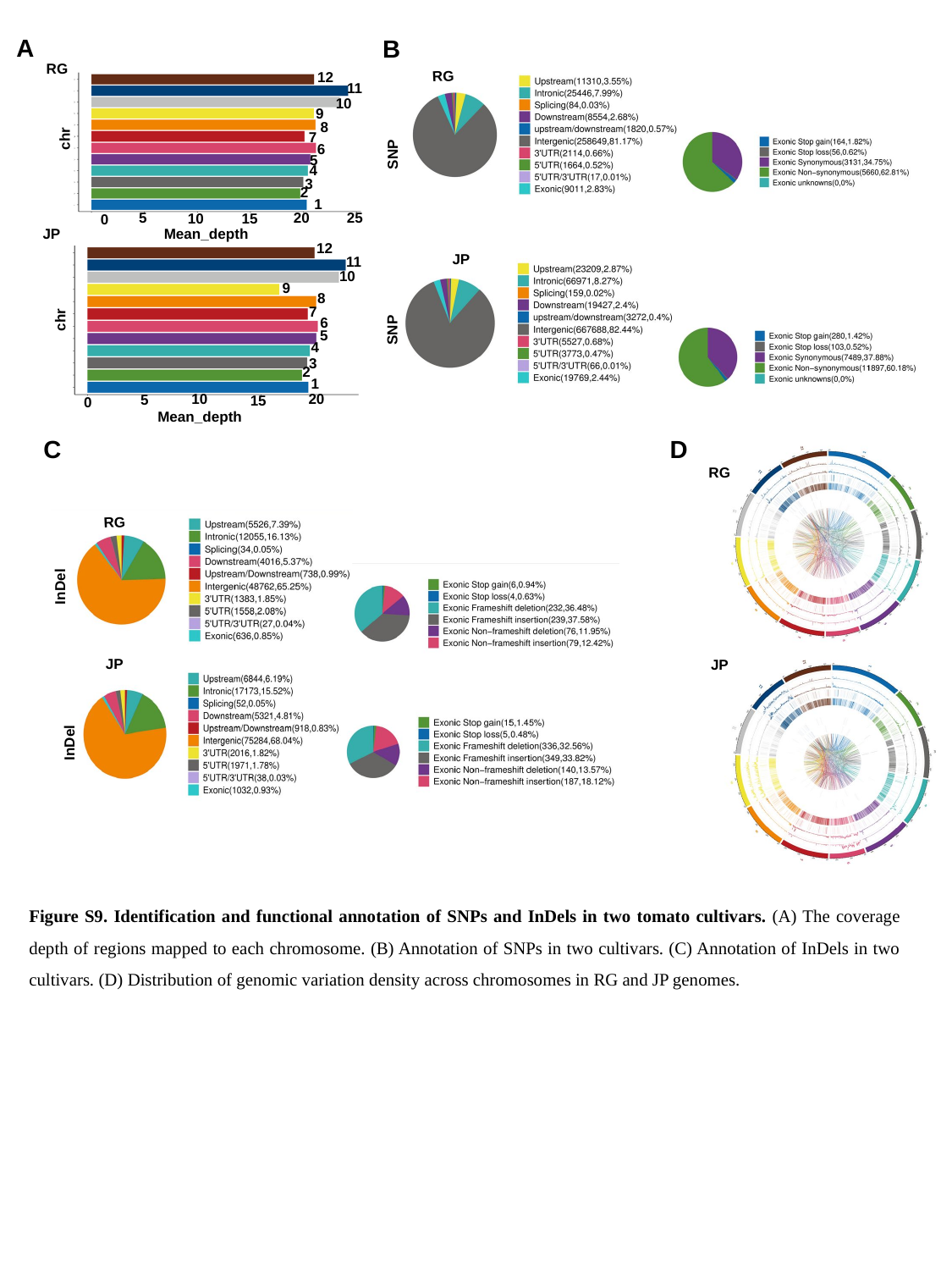

A
B
RG
12
11
10
9
8
7
6
5
4
3
2
1
chr
5
20
25
15
10
0
Mean_depth
RG
SNP
JP
12
11
10
9
8
7
chr
6
5
4
3
2
1
10
20
5
15
0
Mean_depth
JP
SNP
C
D
RG
RG
InDel
JP
JP
InDel
Figure S9. Identification and functional annotation of SNPs and InDels in two tomato cultivars. (A) The coverage depth of regions mapped to each chromosome. (B) Annotation of SNPs in two cultivars. (C) Annotation of InDels in two cultivars. (D) Distribution of genomic variation density across chromosomes in RG and JP genomes.

## Slide 11
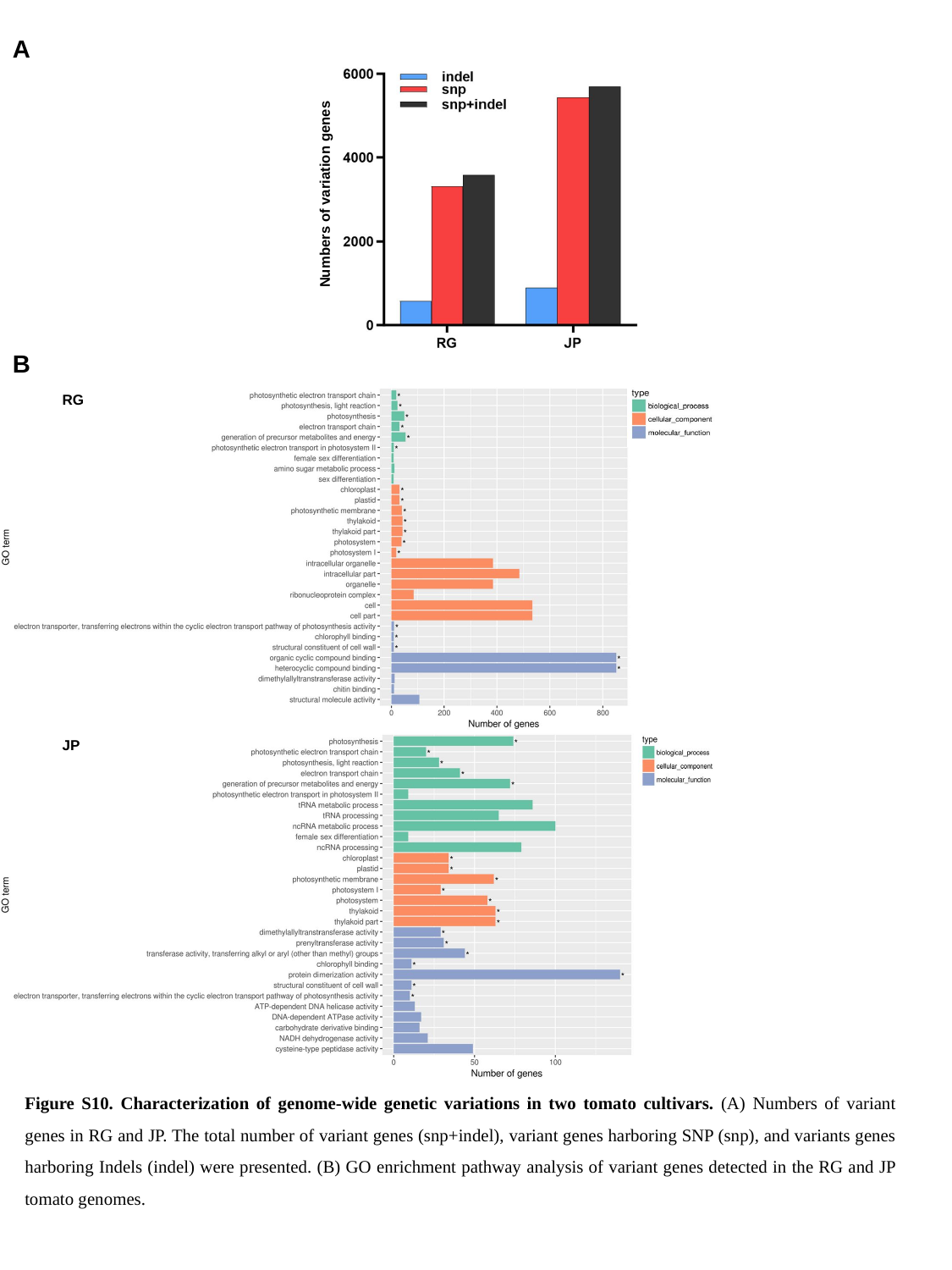

A
Numbers of variation genes
B
RG
JP
Figure S10. Characterization of genome-wide genetic variations in two tomato cultivars. (A) Numbers of variant genes in RG and JP. The total number of variant genes (snp+indel), variant genes harboring SNP (snp), and variants genes harboring Indels (indel) were presented. (B) GO enrichment pathway analysis of variant genes detected in the RG and JP tomato genomes.
